# Mass spectrometry reveals the chemistry of formaldehyde cross-linking in structured proteins

**DOI:** 10.1038/s41467-020-16935-w

**Published:** 2020-06-19

**Authors:** Tamar Tayri-Wilk, Moriya Slavin, Joanna Zamel, Ayelet Blass, Shon Cohen, Alex Motzik, Xue Sun, Deborah E. Shalev, Oren Ram, Nir Kalisman

**Affiliations:** 1 Institute of Life Sciences, The Hebrew University of Jerusalem, Jerusalem, 9190401 Israel; 2 Institute of Chemistry, The Hebrew University of Jerusalem, Jerusalem, 9190401 Israel; 3 Wolfson Centre for Applied Structural Biology, The Hebrew University of Jerusalem, Jerusalem, 9190401 Israel; 4 Department of Pharmaceutical Engineering, Azrieli College of Engineering, Jerusalem, Israel

**Keywords:** Protein-protein interaction networks, Mass spectrometry, Molecular modelling, Chemical tools

## Abstract

Whole-cell cross-linking coupled to mass spectrometry is one of the few tools that can probe protein–protein interactions in intact cells. A very attractive reagent for this purpose is formaldehyde, a small molecule which is known to rapidly penetrate into all cellular compartments and to preserve the protein structure. In light of these benefits, it is surprising that identification of formaldehyde cross-links by mass spectrometry has so far been unsuccessful. Here we report mass spectrometry data that reveal formaldehyde cross-links to be the dimerization product of two formaldehyde-induced amino acid modifications. By integrating the revised mechanism into a customized search algorithm, we identify hundreds of cross-links from in situ formaldehyde fixation of human cells. Interestingly, many of the cross-links could not be mapped onto known atomic structures, and thus provide new structural insights. These findings enhance the use of formaldehyde cross-linking and mass spectrometry for structural studies.

## Abstract (web summary)

Formaldehyde (FA) is a popular cross-linking reagent, but applying it for cross-linking mass spectrometry (XLMS) has been largely unsuccessful. Here, the authors show that cross-links in structured proteins are the product of two FA molecules and identify hundreds of FA cross-links by XLMS in vitro and in situ.

## Introduction

Formaldehyde (FA) has been used as a fixative and preservative for many decades^1,2^. It is reactive toward both proteins and DNA, and forms inter-molecular cross-links between macromolecules^3^, as well as intra-molecular chemical modifications^4,5^. The high reactivity of FA together with its high permeability into cells and tissues has led to its use in numerous applications in biology, biotechnology, and medicine^6^. FA cross-linking of proteins is assumed to involve the formation of a methylene bridge between two proximal amino acids (R^1^-CH_2_-R^2^)^7,8^. However, direct evidence to support this mechanism is sparse. In terms of mass, the methylene bridge adds 12 Da (one carbon atom) to the total mass of the two cross-linked amino acids. Mass spectrometry has confirmed this 12 Da addition to the masses of short linear peptides after FA incubation^5,9–11^. Yet, these studies were not able to identify pairs of peptides that were linked via methylene bridges. Thus, it is unclear whether the observed 12 Da additions were bona fide cross-links or simply local modification of a single peptide.

Another puzzling fact is the lack of reports on the use of FA in the experimental technique of cross-linking coupled to mass spectrometry (XL-MS)^12–14^. In XL-MS, mass spectrometry identifies the protein residues that are linked based on the unique mass of the cross-linker. This information is then used to probe protein interactions^15^ and structures^16^. It seems fair to assume that if the methylene bridge reaction were easy to detect, FA would have been commonly used for in situ XL-MS^17–20^. Yet, we were only able to find reports of FA being used to stabilize protein complexes that were later cross-linked with a different reagent^21,22^. Given this lack of evidence, we hypothesize that FA cross-linking of proteins involves a different chemical mechanism. Identification of cross-linked peptides requires accurate knowledge of the chemical mechanism in order to calculate the mass of the cross-link product. Specifically, a search of mass spectrometry data with an incorrect mass of the adduct will not yield any identifications. Here we conduct an unbiased mass-spectrometric search for the FA adduct that leads to a different reaction product with a mass of 24 Da and not the 12 Da expected. This reaction only occurs in structured proteins (rather than peptides), perhaps explaining why earlier studies did not observe it.

## Results

### FA cross-linking of purified proteins

**Fig. 1:**
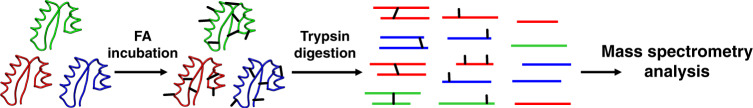
The experimental setup for cross-linking of structured proteins by FA. The tryptic digest consists of a mixture of linear peptides, linear peptides with FA-induced modifications, and cross-linked peptide pairs.

**Fig. 2:**
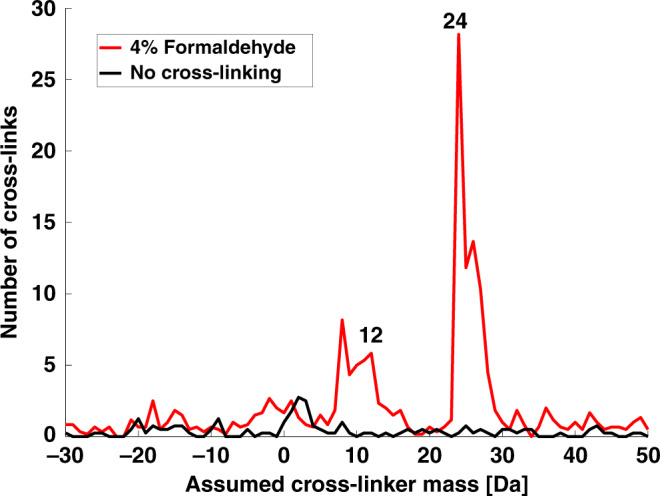
FA cross-linking adds 24 Da to the peptide pairs. Standard search for cross-links in mass spectrometry data from the setup that is described in Fig. 1. The *Y*-axis counts the number of identified cross-links. The search was repeated multiple times, each time assuming a different mass (*X*-axis) being added by the cross-linking reaction to the total mass of the two linked peptides. Overlaid are mass scans from an experiment with high (4%) FA concentration (red) and a control without cross-linking (black). Previously, FA cross-links were thought to be primarily methylene bridges that add 12 Da, but in fact these contribute to very few identifications. Source data are provided as a Source Data file.

We first surveyed the FA cross-linking products that occur within structured proteins by cross-linking a mixture of three purified proteins (bovine serum albumin (BSA), Ovotransferrin, and α-Amylase). The mixture was incubated with FA for twenty minutes, and then quenched, denatured, digested by trypsin into peptides, and analyzed by mass spectrometry (Fig. 1). The general practice to identify a cross-link is by matching the measured mass to a theoretical total mass of the two peptides plus the mass of the cross-linker. Here, we did not limit our search to one predetermined cross-linker mass, but rather scanned through a range of possible masses. Figure 2 shows the number of cross-links that the scan identified for each cross-linker mass that was tested. It was surprising to see that the dominating reaction product adds exactly 24 Da (two carbon atoms) to the total mass of the two peptides. This is different from the 12 Da mass expected under the methylene bridge mechanism^7^. The broadening of the peak, which apparently includes reactions that add 25, 26 and 27 Daltons, is an artifact resulting from incorrect assignment of the mono-isotopic mass by the mass spectrometer (Supplementary Fig. 1). This artifact is common in XL-MS analysis^23,24^ and should not be interpreted as being due to alternative reaction products. We also tested a different brand of FA, which resulted in the same mass-scan profile (Supplementary Fig. 2a).

We find that the 24 Da reaction is not two separate 12 Da reactions occurring in parallel for two reasons: First, while one expects that a lower concentration of FA will show less of the 24 Da reaction and more of the 12 Da reaction, we find that for both high and low concentrations of FA the mass-scan profiles are the same (Supplementary Fig. 2b). Second, ion species corresponding to mass additions of 36 or 48 Da were not observed in Fig. 2, but such species should have occurred according to a parallel cross-linking model.

**Fig. 3:**
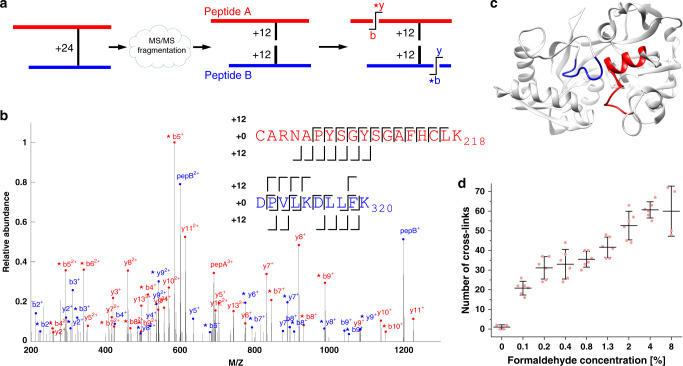
Mass spectrometry characteristics of FA cross-links. **a** The MS/MS fragmentation of a formaldehyde cross-link breaks the ion to its two peptides, each with an additional mass of 12 Da. The two peptides then break a second time to produce b- and y-fragments. **b** The MS/MS spectrum of a cross-link between two peptides of ovotransferrin. pepA and pepB are peaks matching the total mass of the corresponding peptides plus 12 Da. Peaks annotated with *b or *y match the masses of the corresponding b- and y-fragments plus 12 Da. For clarity, only peaks with intensities in the top quartile are shown. The annotation of the full spectrum is shown in Supplementary Fig. 3. **c** The two peptides identified in the above MS/MS spectrum are spatially close to each other in the crystallographic structure of ovotransferrin (PDBid 1OVT^38^). **d** The average number of cross-links identified for each formaldehyde concentration on a mixture of three proteins. Means and standard deviations calculated across three independent experiments, each with two technical replicates. The estimated false-detection rate is 3%. Source data are provided as a Source Data file.

Further support for the uniqueness of the 24 Da reaction is seen in the unusual fragmentation pattern of its MS/MS spectra (Fig. 3a–c). We find that the cross-link is highly susceptible to higher-energy collisional dissociation (HCD), and fragments in which it stayed intact could not be detected. Instead, it breaks symmetrically to give a mass addition of 12 Da on each peptide. Peaks corresponding to the total mass of one of the peptides plus 12 Da were among the most intense in the observed MS/MS spectra. The two peptides then break a second time to yield the standard b- and y-fragments as well as modified b- and y-fragments with an additional 12 Da mass. We find additional evidence for this two-step fragmentation model when we follow the change in fragmentation as a function of the normalized collision energy (Supplementary Fig. 4). Low collision energies are sufficient to break the cross-links, but are insufficient to break the stronger bonds of the b- and y-fragments. The unique fragmentation pattern associated with the 24 Da reaction resembles that of the cleavable cross-linkers frequently used in XL-MS^25^. Yet, an important distinction is the 100% cleavage efficiency of the 24 Da reaction, much higher than observed with other cleavable cross-linking reagents. The unusual fragmentation may partly explain why the 24 Da reaction was not reported in previous FA studies.

With the understanding of the unique properties associated with XL-MS of FA, we designed an analysis application that is tailored specifically to identify the 24 Da reaction and its subsequent MS/MS pattern. The application successfully identified cross-links in the three-protein mixture in a concentration-dependent manner (Fig. 3d). Interestingly, the application could also detect a small number of cross-links corresponding to the 12 Da reaction, but at a ratio of less than 1:7 relative to the 24 Da reaction. Supplementary Data 1 lists an example of the identifications from one such cross-linking experiment. An attempt to analyze the same data with MeroX, an application tailored for cleavable cross-linkers^26^, gave only a third of the identifications (Supplementary Data 2), and these were a subset of our results. The smaller number is caused by certain features of FA cross-linking, such as multiple link sites, that are currently not supported by MeroX.

The modified +12 Da fragments in the MS/MS spectra allowed us to better characterize the amino acids that are most likely to partake in the reaction. To that end, we computationally modified in turn each residue along the cross-linked peptides, and determined which modification site was most compatible with the observed fragmentation pattern. The number of times each amino acid was found to be the most compatible was then normalized by dividing it by the total number of occurrences of that amino acid. This analysis clearly marks lysine and arginine residues to be the most prevalent in the 24 Da reaction (Supplementary Fig. 5). The high reactivity of FA with these two amino acids is fully consistent with previous studies performed on peptides and single amino acids^5,7,10^. However, we note that a third of the identified cross-links involve at least one peptide that does not have a lysine residue. In these particular peptides aspartic acid and tyrosine residues are the most likely to be the linked residues. Interestingly, tyrosine was previously shown to be the third most reactive residue toward FA under certain conditions^5^. We conclude that the majority of FA cross-links occur between lysine or arginine residues, but a significant fraction of cross-links also involve asparagine, histidine, aspartic acid, tyrosine, and glutamine residues.

The fragmentation pattern of the 24 Da reaction does not enable identification of the two residues undergoing cross-linking. As a typical example, the fragmentation pattern of the peptide pair shown in Fig. 3b is consistent with the cross-link occurring on any of the first four residues in the upper (red) peptide. The localization is also ambiguous in the lower (blue) peptide as the first aspartic residue and the middle lysine-aspartic residues are all likely sites for the cross-link given the fragmentation. Therefore, the MS measurement shown in Fig. 3b may actually report a group of isomers of the same two peptides with different cross-link sites on each. This ambiguity usually does not occur with cross-linking reagents with high chemical specificity toward one particular amino acid type. The uncertainty in localizing the cross-link sites prevents the measurement of the exact distance spanned by a FA cross-link. Instead, we estimate the cross-link distance as the minimal Cα–Cα distance between the two peptides on the protein structure. Supplementary Fig. 6 shows the histograms of the minimal distances observed for the cross-links from several FA concentrations, and results from experiments with the cross-linking reagent disuccinimidyl suberate (DSS). This comparison indicates that FA cross-links are on average shorter than those of DSS.

### FA modifications on linear peptides

As a control to the experiments on structured proteins, we incubated the peptide digest from the same three proteins with FA, and analyzed the products by mass spectrometry (Supplementary Fig. 7a). This analysis did not identify any cross-link between a pair of peptides in the digest. Yet, an analysis of single linear peptides found a high abundance of FA-related modifications (Supplementary Fig. 7b, c). Contrary to the cross-links, which adds 24 Da, these modifications are dominated by a reaction that adds 12 Da to the peptides. Just 20 min of incubation with 2% FA, is sufficient to form peptides with a single 12 Da modification at significant numbers. These modifications were nearly absent when the digest was not treated with FA (No XL), and can therefore be attributed to the FA reactivity. Peptides with multiple modifications in parallel (24, 36, 48, and 60 Da) were also frequent, and increased in frequency at longer incubation times. Such modifications are fully consistent with observations of previous mass spectrometry studies of FA effects in peptides^5,9–11^. We conclude that the chemistry of local modifications is fundamentally different from that of long-range cross-linking. Whereas a 12 Da reaction is the most prevalent for local modifications, a 24 Da reaction dominates cross-linking.

### In situ FA cross-linking of human cell cultures

With this clear understanding of the 24 Da cross-linking reaction, we attempt to identify FA cross-links from in situ cross-linking experiments on intact human cells. PC9 adenocarcinoma cells were incubated in 1%, 2%, 3%, 4.5%, or 6% FA solutions for 10 min. After the FA was washed out, the cells were lysed and the protein content prepared for mass spectrometry. We measured 10% of the peptide digest from each FA concentration directly in the mass spectrometer. The other 90% were enriched for cross-linked peptides using SCX^27^, and then measured in the mass spectrometer. Standard proteomics analysis identified in the digests a set of 1692 proteins with medium-to-high abundance. In order to speed up the search for cross-links, we took advantage of the complete dissociation of the FA cross-link during MS/MS fragmentation, which allows matching each peptide to the fragments independently of the other in the pair. An application implementing this strategy analyzed each mass spectrometry run against the database of the 1692 proteins in about 5 min (“Methods”).

Overall, the in situ cross-linking experiments involved 59 data-dependent mass spectrometry runs. The analyses of these runs searched for two separate cross-linker masses: 12 and 24 Da. We then pooled together all the identifications from these analyses into a non-redundant list of 559 cross-links (Supplementary Data 3). The false-detection rate for this list of cross-links was estimated to be 3% of the entire list, and 16% of the inter-protein list. The false-detection rate estimation was based on decoy analysis that spiked the search database with reversed sequences (“Methods”). The 24 and 12 Da cross-linking reactions accounted for 74 and 26% of the cross-links, respectively. This reaffirms the dominance of the 24 Da reaction in FA cross-linking also in the case of in situ FA applications. Interestingly, the 12 Da reaction is more prevalent in situ than it was for the mixture of purified proteins, possibly reflecting influences of the cellular environment on its efficiency.

**Fig. 4:**
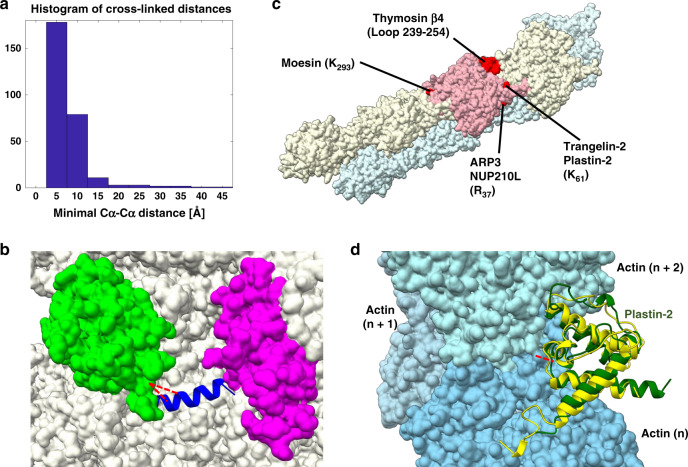
In situ FA cross-linking of human PC9 cells in culture. **a** Histogram of the distances spanned by in situ cross-links in cases that could be mapped onto solved atomic structures. The PDB IDs used for calculating these distances are listed in Supplementary Data 3 for each cross-link. **b** Docking model of the binding helix of βNAC (blue) on the outer surface of the S80 ribosome (PDBid 6EK0^39^). The docking was constrained by two cross-links (red) to ribosomal protein L22 (green). Ribosomal protein L31 (magenta) was shown previously to interact with βNAC as well^29^. **c** A view of the actin filament (PDBid 6D8C^31^), with one of its monomers marked in pink. Cross-links between actin and several of its regulators all map to the outer surface of the actin filament (red). **d** Docking model (green) of the CH3 domain of plastin-2 onto the actin filament. The docking was constrained by a single cross-link (red) between plastin-2 and actin. The model is similar (3.2 Å RMSD) to a recent cryo-EM structure (PDBid 6D8C^31^) of the homologous protein filamin A (yellow), which was assembled on the filament in vitro. Source data are provided as a Source Data file.

The identified cross-links occur within a subset of 276 proteins that are of relatively high abundance in the PC9 cell line^28^. This is expected because we did not enrich for any particular protein. Encouragingly, the cross-linked proteins originate from the nucleus (histones), cytoplasm (ribosomes and TRiC/CCT), mitochondria (HSP60), and endoplasmic reticulum (BiP), indicating that the FA has reached most cellular compartments. We could map 280 of the cross-links onto solved atomic structures. Figure 4a shows the histogram of the minimal Cα–Cα distances spanned by these cross-links. The histogram includes only cross-links between two peptides that are not consecutive along the protein sequence. The FA cross-links fit the atomic structures well, having a minimal Cα–Cα distance below 25 Å for 97% of them (272 cases).

Of the 559 cross-links, 90 (16%) are inter-protein (between two different proteins in a complex) and the rest are intra-protein (within the same protein polypeptide). A subset of 28 inter-protein cross-links had no corresponding atomic structures, but they showed strong indications of being true positives. All had good fragmentation of both peptides (20 fragments or more on the weakest peptide), and most were previously reported to be part of a protein complex (Table 1). These cross-links provide structural data—of in situ origin—on the relevant interactions. Particularly, each cross-link narrows down the interaction site to the vicinity of the two linked peptides.

**Table 1:** Inter-protein cross-links that provide new in situ structural information.

System	Protein 1	Protein 2	Peptide 1	Peptide 2	Reference for the interaction
Ribosome + NAC	BTF3 (βNAC)	RL22	E_47_TIMNQEKLAK_57_	Y_114_FQINQDEEEEEDED_128_-Cterm	^40^
	BTF3 (βNAC)	RL22	L_55_AKLQAQVR_63_	Y_114_FQINQDEEEEEDED_128_-Cterm	^40^
Actin	Actin	Transgelin-2	D_51_SYVGDEAQSKR_62_	Y_103_GINTTDIFQTVDLWEGK_120_	^41^
	Actin	Arp3	A_29_VFPSIVGRPR_39_	V_51_MKGVDDLDFFIGDEAIEKPTYATK_75_	^42^
	Actin	Plastin-2	D_51_SYVGDEAQSKR_62_	F_473_SLVGIGGQDLNEGNR_488_	^43^
	Actin	Moesin	K_293_DLYANNVLSGGTTMYPGIADR_314_	T_539_ANDMIHAENMRLGR_553_	^44^
	Actin	Thymosin β-4	S_239_YELPDGQVITIGNER_254_	E_33_TIEQEKQAGES_44_-Cterm	^45^
	Actin	NUP210L	A_29_VFPSIVGRPR_39_	A_92_VLIAESTQPIR_103_	New
	Actin-Beta	Actin- cytoskeletal	I_192_LTERGYSFTTTAER_206_	V_98_APEEHPTLLTEAPLNPKANR_118_	^46^
	α-Actinin 4	α-Actinin 1	D_758_AKGISQEQMQEFR_771_	L_345_RLSNRPAFMPSEGR_359_	New
Condensin	Smc4	Smc2	S_491_VNEARSK_498_	N_407_DISKAQTEAK_417_	^47^
TMED	TMED9	TMED5	Q_170_LVEQVEQIQK_180_	L_153_EDILESINSIKSR_166_	^48^
HNRP	HNRPD	HNRPU	L_130_DPITGRSR_138_	A_559_PQCLGKFIEIAAR_572_	^46^
—	ABCF2	OLA1	E_135_VPIPEHIDIYHLTR_149_	Y_282_LEANMTQSALPKIIK_297_	New

We highlight two subsets of cross-links, which were employed for constrained docking. The first subset involves the binding site of the nascent polypeptide-associated (NAC) complex on the ribosome. Previously, Pech et al.^29^ showed that a conserved region in βNAC, which is predicted to form an α-helix, is binding with the ribosome. Two in situ cross-links cover this sequence region, and link it to the C-terminal of ribosomal protein L22. We applied PatchDock^30^ with the restraints of the cross-links, to dock a model of that region onto the ribosome. The best scoring model (Fig. 4b) was close to two ribosomal proteins L22 and L31, a binding mode that is consistent with previous in vitro evidence showing βNAC to also interact with L31^29^.

A larger subset of cross-links mapped the interaction sites of several actin regulators onto the outer surface of the actin filament (Fig. 4c). This is consistent with their functions in regulation of bundling and bifurcation of the filaments. We performed all-atom docking onto the actin filament of plastin-2, for which a reliable homology model of the actin-binding CH domain could be built. This docking was restrained by the cross-link between actin and plastin-2. Remarkably, the model that ranked third by its PatchDock score had a 3.2 Å deviation from a recent cryo-EM structure of filamin A (Fig. 4d), which is homologous to plastin. The available cryo-EM structures^31,32^ were determined from in vitro reconstruction of actin filaments with a large excess of filamin A. Thus, our docking result provides in situ support for the relevance of the cryo-EM structure. Moreover, it suggests that the binding of filamin and plastin to the actin filament are very similar.

In contrast to the cross-links in Table 1, a subset of nine inter-protein cross-links had two different indications of being false positives. First, they had marginal MS/MS fragmentation evidence (14–19 fragments on the weaker peptide in the pair). Second, the two cross-linked proteins had never been reported in the literature to be interacting. Assuming that all the intra-protein cross-links are correct, then these nine cross-links are the only false positives in the entire list. As they comprise 1.6% of the list (9 out of 559), this is in accord with our a priori estimation of the false-detection rate.

## Discussion

We have established four features of long-range FA cross-links in proteins. First, they occur only in structured proteins. Hence, the reliance of previous studies on peptide assays incorrectly classified the prevalent 12 Da modification as a cross-link. Second, the dominant cross-linking reaction involves two carbon atoms (24 Da) and not one. Third, these cross-links are very labile and cleave completely under MS/MS fragmentation. Finally, the most intense MS/MS fragmentation products carry an unusual 12 Da modification. We believe that all these factors have contributed to the fact that the chemistry of the long-range FA cross-link has not been characterized correctly.

**Fig. 5:**
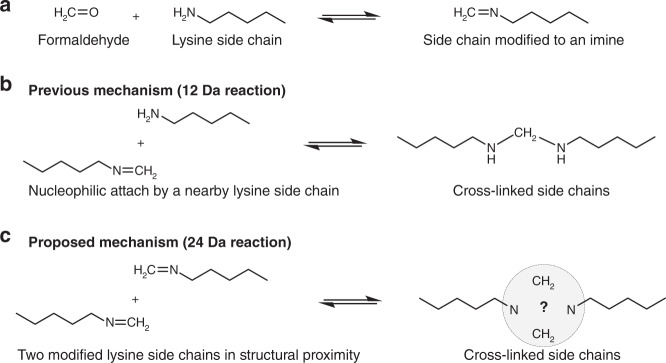
Proposed mechanism for the 24 Da cross-linking reaction. The mechanism is demonstrated on two lysine side chains. **a** Formaldehyde modifies the primary amine of a lysine side chain into an imine. These modifications are well established by mass spectrometry. **b** Previously, it was thought that the cross-link is a methylene bridge that forms between a lysine side chain and a nearby imine modification. **c** Our data strongly suggest that formaldehyde cross-links are mainly the dimerization product of two modified side chains. The dimerization step is presumably slow and can occur only in structured proteins, in which the two side chains are close and nearly stationary relative to each other. The current data cannot conclusively determine the chemical structure at the linkage site.

In light of the findings, we suggest the following mechanism of FA cross-linking (Fig. 5). The reaction starts with the accepted imine formation on the side chains of lysines. The imine formation is in accord with the prevalent 12 Da modification that others and we have observed on peptides and proteins. However, the cross-link itself forms by a dimeric interaction of two imines^33^. This symmetric formation is compatible with three observations. First, it explains the symmetrical cleavage of the link under MS/MS fragmentation. Second, if one assumes that the imine modification is only mildly reactive, then it is clear why cross-linking occurs only in structured proteins: the stable structure of the protein keeps the modifications in proximity for sufficient time for cross-linking to occur. Third, the dimerization is consistent with the known reversibility of FA cross-linking, which implies that all steps of the mechanism are reversible. In particular, the MS/MS spectra clearly demonstrate the full reversal of the last dimerization step by the introduction of mild collision energy.

In Fig. 5, the cross-linking mechanism is exemplified on two lysine side chains, but FA cross-linking does not necessarily require two lysines. Indeed, for many of the in situ cross-links (Supplementary Data 3) one of the peptides has no lysine residues. Therefore, the hypothesized model would have to be revised for cross-linking in the more general case. The current data cannot conclusively determine what is the chemical structure of the linkage site. One possibility is that the two imines undergo cycloaddition to form a 1,3-diazetidine linkage. Such a strained ring structure would be consistent with the tendency of the link to break completely under HCD fragmentation. Nonetheless, other chemical structures are equally possible and efforts to better characterize the linkage site by NMR are ongoing.

In our experience, FA is not a more potent reagent compared with reagents based on NHS-esters. Yet, it has several advantages, notably its solubility and proven ability to penetrate cells and tissues rapidly. This makes FA an attractive reagent for in situ XL-MS, which is currently not as developed as XL-MS applications on purified protein solutions or lysates. We believe that the findings of this work will now allow for a much wider use of FA for in situ XL-MS experiments.

## Methods

### Cross-linking of the three-protein mixture

A mixture solution of three purified proteins was prepared by reconstituting lyophilized protein powder in PBS (all reagents were purchased from Sigma unless noted otherwise). The proteins were bovine serum albumin (BSA), Ovotransferrin, and α-Amylase with respective final molarity in the mixture of 10, 10, and 20 µM. Each cross-linking experiment occurred in 108 µL of solution comprising a total protein mass of 260 µg. In most experiments we cross-linked with a formalin solution (37% FA and 10% methanol) from Sigma (product number F8775). We also tested formalin with the same composition from another brand (DAEJUNG chemicals, Korea, product number 4044-4400). The formalin was incubated with the protein mixture at the desired FA concentration and the cross-linking reaction occurred at room temperature under gentle agitation. The cross-linking incubation time was 20 min. The cross-linking reaction was quenched by addition of ammonium bicarbonate to a final concentration of 0.5 M for 10 min before proceeding to mass spectrometry preparation. The results of each experimental condition are an average of six mass spectrometry runs from three experimental replicates, each with two technical replicates.

### Cross-linking of digest from the three-protein mixture

Peptide digest was prepared from the three-protein mixture by trypsin digestion as described in the Mass spectrometry subsection ahead. The peptides were desalted on SepPak C18 column (Waters), eluted, dried in SpeedVac, and reconstituted in PBS. FA was added to a concentration of 2% and the incubation time was either 20 min, 2 h, or 24 h. The solution was quenched by addition of ammonium bicarbonate to a final concentration of 0.5 M for 10 min. The peptides were desalted on C18 stage tips and eluted for mass spectrometry analysis. The results of each incubation time are an average of two experimental replicates, each with two technical replicates.

### In situ cross-linking of PC9 cells

Human lung cancer cell line PC9 (ECACC, catalog No. 90071810) were seeded in Dulbecco’s modified Eagle’s medium, and were supplemented with 1× penicillin–streptomycin (Gibco Invitrogen) and 10% fetal bovine serum (Biological Industries) at 37 °C under 5% CO_2_/95% air. The cells were grown to 80% confluency in 10-cm plates. The growth media was removed and the cells washed three times with 3 ml of warm PBS buffer. We added to each plate 2 ml of PBS with FA at different concentrations: 1, 2, 3, 4.5, or 6%. The cells were incubated with FA for 15 min at 37 °C, and then washed three times with cold PBS to remove the FA. We incubated the cells with hypertonic buffer (50 mM HEPES pH = 7.5, 500 mM NaCl, 0.5 mM EDTA, 0.0005% Tween20) for 15 min, and then scraped the cells from the plate. The cells were centrifuged at 4 °C and the supernatant was discarded. The cell pellet was resuspended for 15 min with hypotonic buffer (above buffer without NaCl), and then further lysed with sonication (5 s on, 25 s off, 5 times, 50% amplitude). The cell lysate was centrifuged at 4 °C and the supernatant was collected. The lysate was processed by the filter-aided sample preparation protocol^34^ in order to remove the detergent and nucleic acids prior to the mass spectrometry analysis.

### Enrichment by strong cation exchange (SCX) chromatography

We followed the SCX protocol by Klykov et al.^27^. Briefly, desalted peptide digest was dried in SpeedVac and reconstituted in 50 μl of buffer A (20% Acetonitrile, formic acid titrated to pH of 3.0). Separation was performed with an Äkta Pure system on a 100 × 1.0 mm PolySULFOETHYL A SCX column (PolyLC, USA) using a gradient of buffer B (20% Acetonitrile, 0.5 M NaCl, formic acid titrated to pH of 3.0) and 100 μl fractions. Fractions corresponding to NaCl concentrations of 100 mM and higher were desalted and used for mass spectrometry analysis.

### Mass spectrometry

The proteins were precipitated in acetone at −80 °C for 1 h followed by centrifugation at 10,000 × *g*. The pellet was resuspended in 20 μl of 8 M urea with 10 mM DTT. After 30 min, iodoacetamide was added to a final concentration of 50 mM and the alkylation reaction proceeded for 30 min. The urea was diluted by adding 200 μl of digestion buffer (25 mM TRIS pH = 8.0; 10% acetonitrile), trypsin (Promega) was added at a 1:100 protease-to-protein ratio, and the protein was digested overnight at 37 °C under agitation. Following digestion, the peptides were desalted on C18 stage tips and eluted by 55% acetonitrile. The eluted peptides were dried in a SpeedVac, reconstituted in 0.1% formic acid, and measured in the mass spectrometer. The samples were analyzed by a 120 min 0–40% acetonitrile gradient on a liquid chromatography system coupled to a Q-Exactive Plus mass spectrometer (Thermo). We were careful not to raise the temperature of the sample above 40 °C through all the preparation stages (alkylation, digestion, desalting, and in the analytical column of the LC) in order not to break the FA cross-links. The RAW data files from the mass spectrometer were converted to MGF format by Proteome Discoverer (Thermo), which was the input format for our analysis applications. The method parameters of the run were as follows: data-dependent acquisition; Full MS resolution 70,000; MS1 AGC target 1e6; MS1 Maximum IT 200 ms; Scan range 450–1800; dd-MS/MS resolution 35,000; MS/MS AGC target 2e5; MS2 Maximum IT 300 ms; Loop count Top 12; Isolation window 1.1; Fixed first mass 130; MS2 Minimum AGC target 800; HCD energy (NCE) 26;Charge exclusion: unassigned,1,2,3,8,>8; Peptide match—off; Exclude isotope—on; Dynamic exclusion 45 s.

### Scanning for the mass of the cross-linking reaction

We modified our analysis application, FindXL^35^, so that it ran multiple times, each time with a different cross-linker mass. We scanned all the integer masses from −30 to 50 Da. FindXL exhaustively enumerates all the possible peptide pairs and compare them to the measured MS/MS events in search of matches that fulfill the criteria below. The search parameters were as follows: Sequence database—the sequences of BSA, Ovotransferrin, and α-Amylase; Protease—trypsin, allowing up to three miscleavage sites; Fixed modification of cysteine by iodoacetamide; Variable modification of methionine by oxidation; Cross-linking can occur on any residue type; Cross-linker is non-cleavable; MS/MS fragments to consider—b-ions and y-ions as well as b-ions and y-ions with the additional mass of the second peptide and the cross-linker; MS^1^ tolerance – 6 ppm; MS^2^ tolerance—8 ppm.

A cross-link was identified as a match between a MS/MS event and a peptide pair if it fulfilled four conditions: (1) The mass of the precursor ion is the same as the expected mass of the cross-linked peptide pair within the MS^1^ tolerance; (2) At least four MS/MS fragments (within the MS^2^ tolerance) were identified on each peptide; (3) The fragmentation score of the cross-link (defined as the number of matching MS/MS fragments divided by the combined length of the two peptides) is 0.6 or higher; (4) The peptides are not overlapping nor consecutive in the protein sequence. The purpose of the fourth criterion is to count only cross-links that span a long range on the primary structure.

### Identifying the amino acids involved in the 24 Da reaction

The identified cross-links from all the replicates involving 2 and 4% FA cross-linking were pooled together for this analysis. For each cross-link, we analyzed the two peptides independently of each other. For each peptide, we computationally modified (added 12 Da) in turn to each residue. We then determined which residue position was most compatible with the MS/MS fragmentation pattern (highest number of fragments that can be assigned by the modified peptide at 8 ppm tolerance). The number of times each amino acid was found to be the most compatible was then normalized by dividing it by the total number of occurrences of that amino acid in all the peptides (normalized count).

### Identifying linear peptides with modifications

The identification of modifications formed by FA on linear peptides was based only on matching the mass of the precursor ion (i.e., MS^1^) to the theoretical mass of the peptide+modification. This approach was taken because of insufficient knowledge as to where these modifications occur, or how they affect the MS/MS fragmentation. To make the identification more stringent, we set a very narrow tolerance of 1 ppm on the match between the measured and theoretical mass of the peptide plus the modification. Of note, with such a narrow tolerance we did not find any ambiguous cases in which the measured mass could be assigned to more than one peptide. We ran the analysis eight times, each time searching for a different modification: 0.0 (no modification), 12.0, 24.0, 36.0, 48.0, 60.0, 57.0215 (off-target alkylation), and 15.9949 (oxidation) Da. The estimate of the relative abundance of each modification was calculated as the ratio between the number of identified peptides with that modification and the number of identified peptides without modification (0.0 Da). Other search parameters were: Sequence database—the sequences of BSA, Ovotransferrin, and α-Amylase; Protease—trypsin, allowing up to three miscleavage sites; Fixed modification of cysteine by iodoacetamide. Methionine oxidation was not considered.

### Cross-link identification in a small set of proteins

This analysis application exhaustively enumerates all the possible peptide pairs, and compare them to the measured MS/MS events in search of matches that fulfill the criteria below. The search parameters were as follows: Sequence database—the sequences of BSA, Ovotransferrin, and α-Amylase; Protease—trypsin, allowing up to three miscleavage sites; Fixed modification of cysteine by iodoacetamide; Variable modification of methionine by oxidation; Cross-linking can occur on any residue type; Cross-linker is always cleaved; MS/MS fragments to consider: b-ions, y-ions, *b-ions (b-ions plus 12.0 Da), and *y-ions (y-ions plus 12.0 Da); MS^1^ tolerance—6 ppm; MS^2^ tolerance—8 ppm; Cross-linker mass—one of three possible masses: 24.0, 25.00335, and 26.0067. The three cross-linker masses were considered in turn in the calculation of the theoretical mass of the two cross-linked peptides. These masses address the incorrect reporting of the mono-isotopic mass (Supplementary Fig. 1).

A cross-link was identified as a match between a measured MS/MS event and a peptide pair if it fulfilled five conditions: (1) The mass of the precursor ion is within the MS^1^ tolerance of the theoretical mass of the linked peptide pair (with either of the three possible cross-link masses); (2) At least four modified MS/MS fragments (*b and *y) were identified within the MS^2^ tolerance on each peptide; (3) The fragmentation score of the cross-link (defined as the number of all matching MS/MS fragments divided by the combined length of the two peptides) is 1.0 or higher; (4) The peptides are not overlapping in the protein sequence; (5) There is no other peptide pair or linear peptide that match the data with equal or better fragmentation score.

Given the small size of the sequence database, we estimated the false-detection rate in the following way. The analysis of data from the 4% FA experiment was repeated ten times with an erroneous cross-linker mass of 61.0, 62.0, 63.0, … 70.0 Da. This led to fragmentation scores that were much lower than the scores obtained with the correct cross-linker mass. On average, 2 erroneous cross-links had a fragmentation score above 1.0 in each decoy run, whereas runs with the correct cross-linker mass (24.0 Da) identified ∼60 cross-links above the 1.0 score. We therefore estimate the false-detection rate to be 2 in 60 cross-links or ∼3%.

### Cross-link identification in a large set of proteins

This application relied on the complete cleavage of the FA cross-links in order to separately assign a MS/MS fragmentation score to each peptide. This division allows for a practical run time of *O(n)* with suitable preprocessing. The search parameters were as follows: Sequence database—comprising the 1692 human proteins that were identified in the samples. Note that runs on the full human proteome (20,000 proteins) are possible, but take up to 4 h; Protease—trypsin, allowing up to two miscleavage sites; Fixed modification of cysteine by iodoacetamide; Cross-linking can occur on any residue type; Cross-linker is always cleaved; MS/MS fragments to consider: b-ions, y-ions, *b-ions (b-ions plus 12.0 Da), and *y-ions (y-ions plus 12.0 Da); MS^1^ tolerance – 4.2 ppm; MS^2^ tolerance – 6.5 ppm; Cross-linker mass—one of five possible masses: 24.0, 25.00335, 26.0067, 12.0, and 13.00335 Da. All of these masses were considered in turn in the calculation of the theoretical mass of the two cross-linked peptides. The five masses address the incorrect reporting of the mono-isotopic mass (Supplementary Fig. 1), as well as the much less frequent 12 Da reaction.

A cross-link was reported if it fulfilled four conditions: (1) The mass of the precursor ion is within the MS^1^ tolerance of the theoretical mass of the cross-linked peptide pair (with any of the five cross-link masses); (2) Each peptide had at least 19 MS/MS fragments (b, y, *b and *y) within the MS^2^ tolerance, OR its fragmentation score (defined as the number of matching MS/MS fragments divided by its length) was 1.8 or higher; (3) The peptides are not overlapping in the protein sequence; (4) There is no other peptide pair or linear peptide that match the data with equal or better fragmentation score.

To estimate the false-detection rate of the reported list of cross-links, we spiked the sequence database with a decoy set comprising some of the sequences in reverse. The proteins used for the decoys were chosen randomly and their number is user defined. In the case of the PC9 lysate, the number of decoy sequences was set to 1/15 the total number of sequences. We therefore estimate the number of false positives in the cross-link list to be 15 times the number of cross-links that include a reverse decoy peptide.

### Computational docking

Docking was performed with PatchDock^30^. The cross-link was implemented as distance constraints that must be under 12 Å in accepted models. Homology models of βNAC and Plastin-2 were generated by HHPred^36^.

### Reporting summary

Further information on research design is available in the Nature Research Reporting Summary linked to this article.

## Supplementary information

Supplementary Information

Peer Review File

Description of Additional Supplementary Files

Supplementary Dataset 1

Supplementary Dataset 2

Supplementary Dataset 3

Supplementary Dataset 4

Supplementary Dataset 5

Reporting Summary

## Data Availability

The mass spectrometry data have been deposited to the ProteomeXchange Consortium via the PRIDE^37^ partner repository with the dataset identifier PXD015435. Source data are provided with this paper. All other data are available from the corresponding author on reasonable request.

## Source data

Source Data

## Code availability

A standalone analysis application for identification of formaldehyde cross-links is available at https://github.com/Kalisman-Lab/Search_Formaldehyde_Cross-links. The underlying source code in Java is available at https://github.com/Kalisman-Lab/Search_Formaldehyde_Cross-links_Source_Code.
