# Supplementary material for: Mass spectrometry reveals the chemistry of formaldehyde cross-linking in structured proteins
Source: Nat Commun. 2020 Jun 19;11:3128. doi: 10.1038/s41467-020-16935-w (PMC7305180; doi:10.1038/s41467-020-16935-w)
Supplement: Supplementary file 1 — Supplementary Information [file 41467_2020_16935_MOESM1_ESM.pdf]

Supplementary Information

Mass spectrometry reveals the chemistry of formaldehyde cross-linking in structured proteins.

Tayri-Wilk, et al.

Supplementary Figures

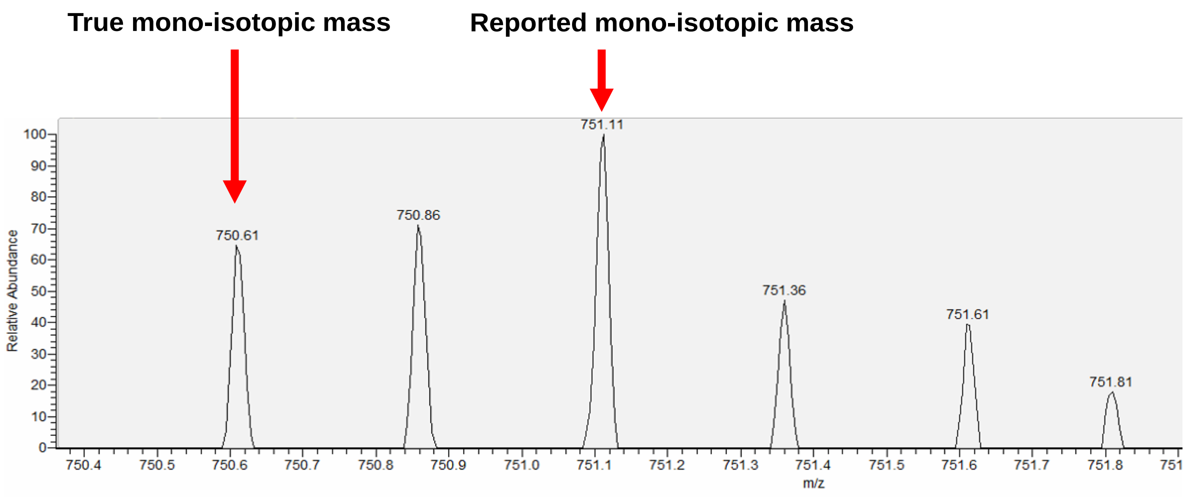

Supplementary Figure 1. Errors in the reported mono-isotopic mass. The most intense peak in the isotope series of cross-linked peptides is usually not the mono-isotopic mass. Consequently, the mass-spectrometer often incorrectly report a mass that is approximately 1, 2, or even 3 Da heavier than the true mass (these values being multiples of the mass difference between Carbon-13 and Carbon-12). In Q-Exactive Plus mass spectrometers, which were used in this work, this incorrect assignment of the mono-isotopic mass occurs for about half of the cross-link identifications. The example here is the isotopic series of an identified cross-link for which the reported mono-isotopic mass was 2 Da heavier than the true mass. This is the cause for the peak broadening to the right of the 24 Da reaction in Figure 2. In this work, we address this artifact by expanding the search to matches with either the reported mass, the mass minus 1.003 Da, or the mass minus 2.006 Da.

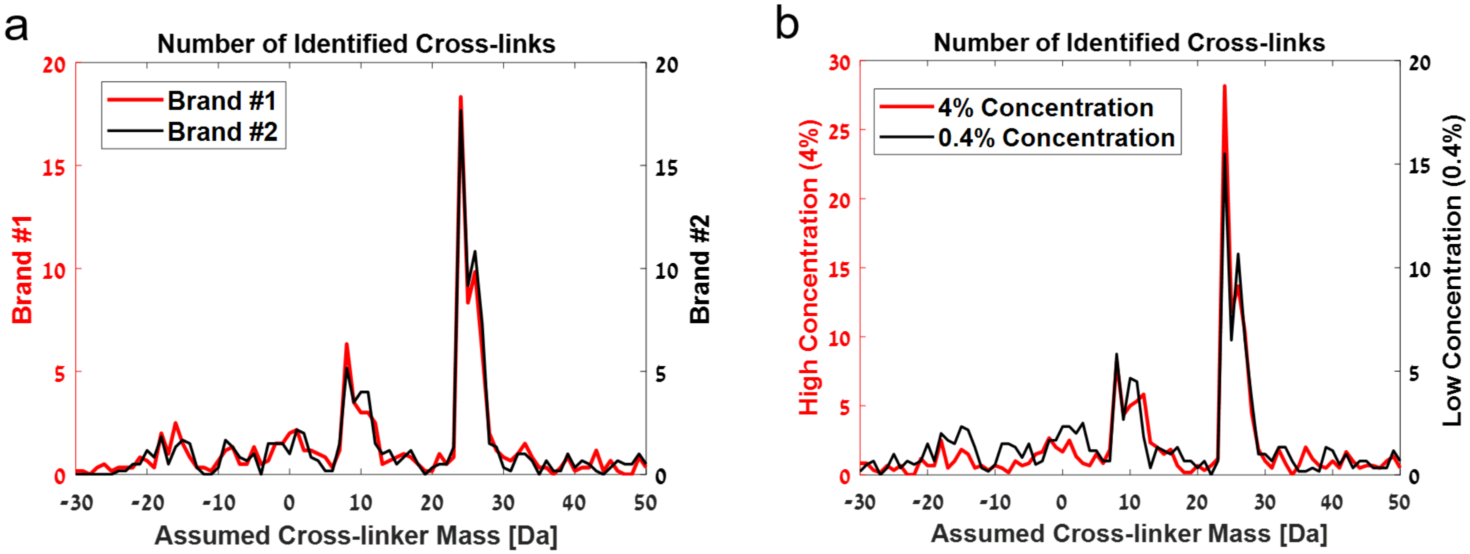

Supplementary Figure 2. The dominance of the 24 Da peak is not affected by the brand or concentration of formaldehyde (FA). (a) Overlaid mass scans (as described in Fig. 2) for data from cross-linking experiments that used two different brands of FA. Both brands gave essentially the same profile. The FA concentration was 1.3%. Brand #1 is from Sigma (product number F8775). Brand #2 is from DAEJUNG chemicals (product number 4044-4400). **(b)** Overlaid mass scans for data from cross-linking experiments that used two different concentrations of FA. Both concentrations gave essentially the same profile, albeit the lower concentration gave less cross-links (compare scales of left and right Y-axes).

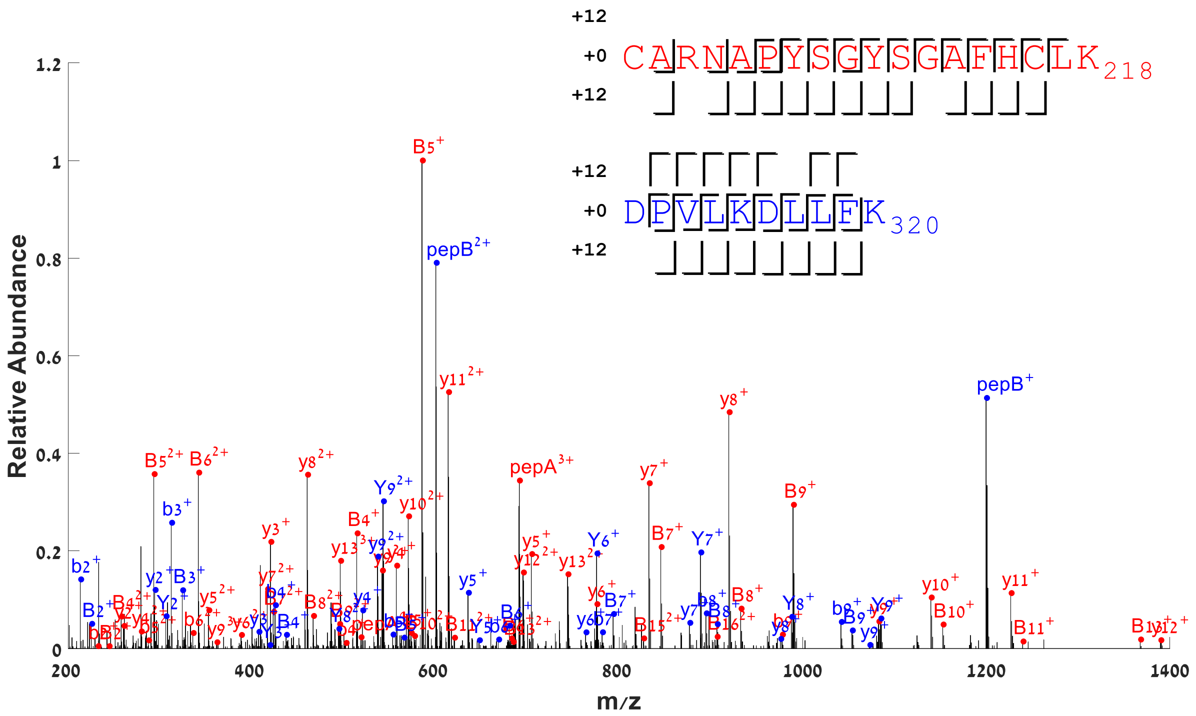

Supplementary Figure 3. The full annotated MS/MS spectrum of two cross-linked peptides from ovotransferrin (relating to Figure 3b). pepA and pepB are peaks matching the total mass of the corresponding peptides plus 12 Da. Peaks annotated with *b or *y match the mass of the corresponding b or y-fragments plus 12 Da.

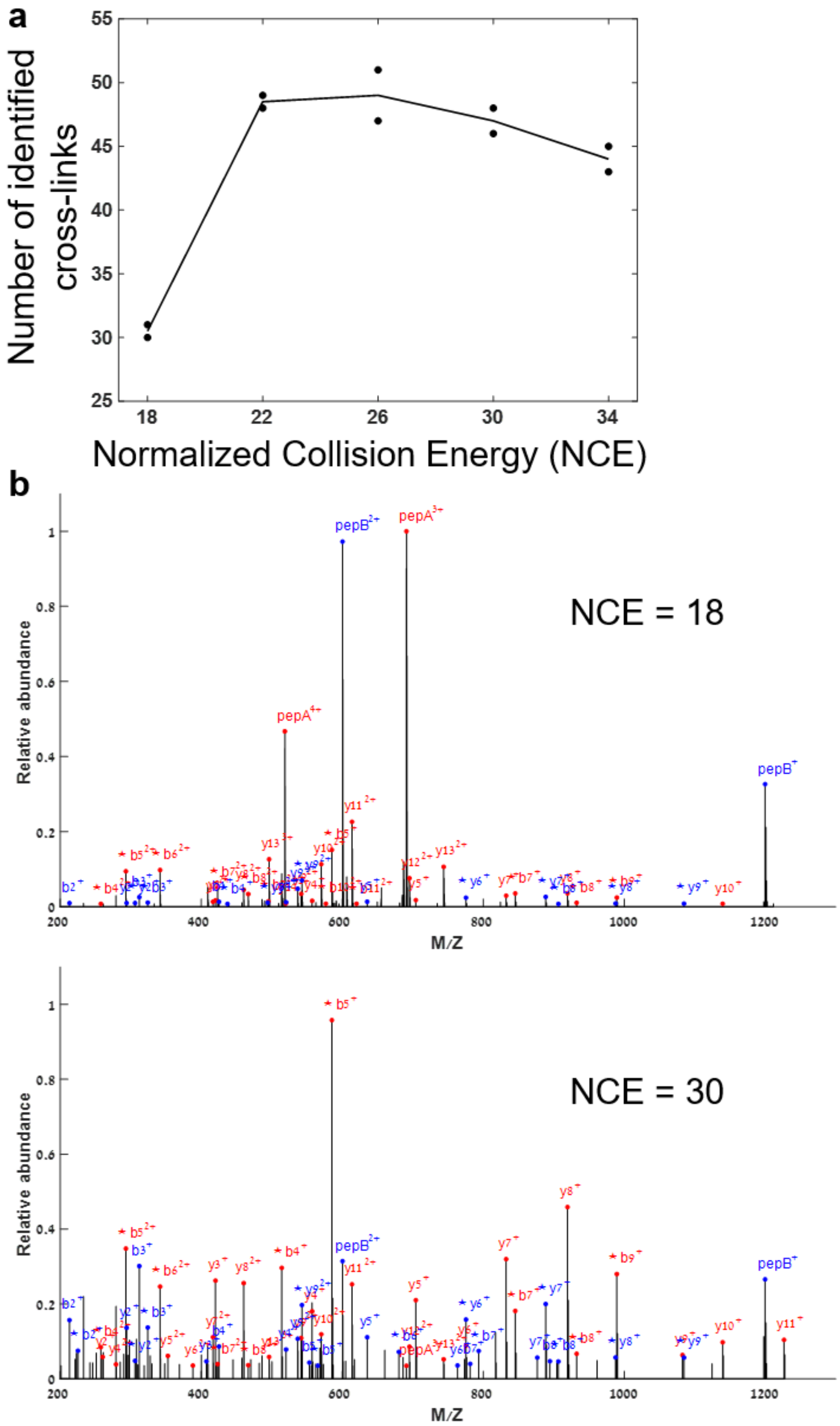

Supplementary Figure 4. Effects of the Normalized Collision Energy (NCE). (a) The optimal NCE for identification of cross-links was determined to be 26. Shown are two experimental replicates (black spheres) measuring a series of HCD energies on a sample of 4% formaldehyde cross-linking of the three-protein mixture. A line through the average values is set to guide the eye. **(b)** Two MS/MS spectra of the same cross-linked ion (CARNAPYSGYSGAFHCLK$_{218}$ and DPVLKDLLFK$_{320}$ from ovotransferrin) measured at two different NCE values. Note that the high peaks corresponding to the two intact peptides plus 12 Da (pepA and pepB) at NCE=18 are greatly reduced at the higher NCE.

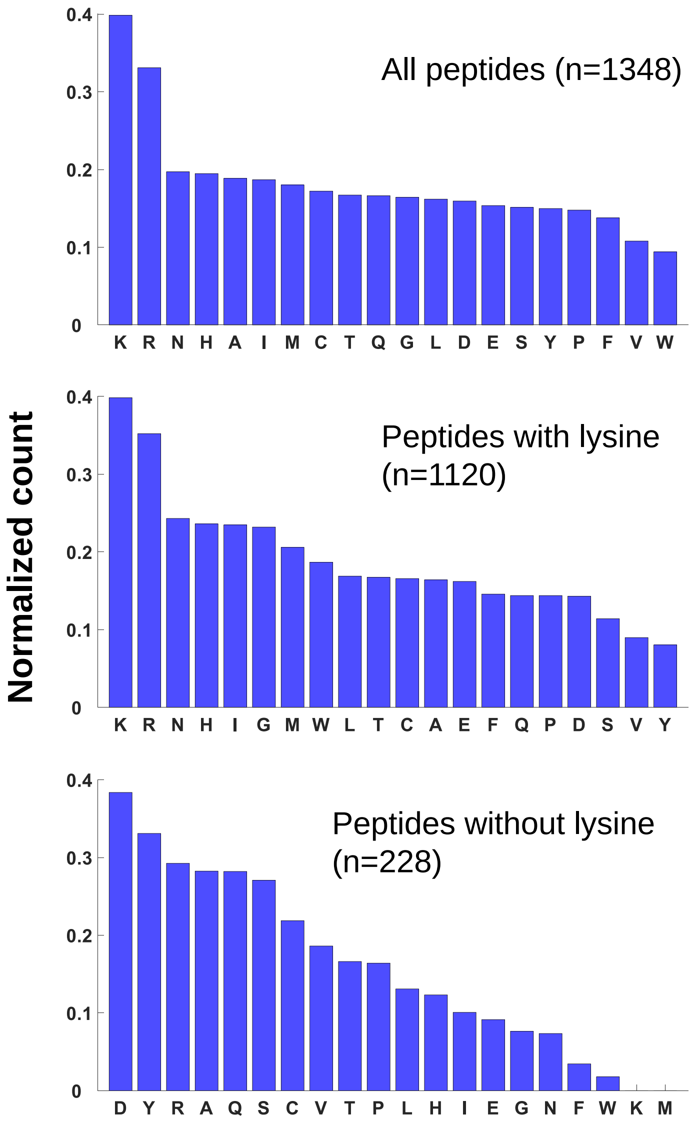

Supplementary Figure 5. The propensity of different amino acids to participate in the 24 Da cross-link. For each peptide in each cross-link, we mark the residue on which a 12 Da modification would be the most compatible with the MS/MS fragmentation. The normalized count is the total number of times a certain amino acid type was the most compatible divided by the total number of occurrences of the same amino acid in all the peptides. The analysis on all the peptides (top plot) was also repeated on two subsets: peptides that have at least one lysine residue (middle plot), and peptides that have no lysine residues (bottom plot).

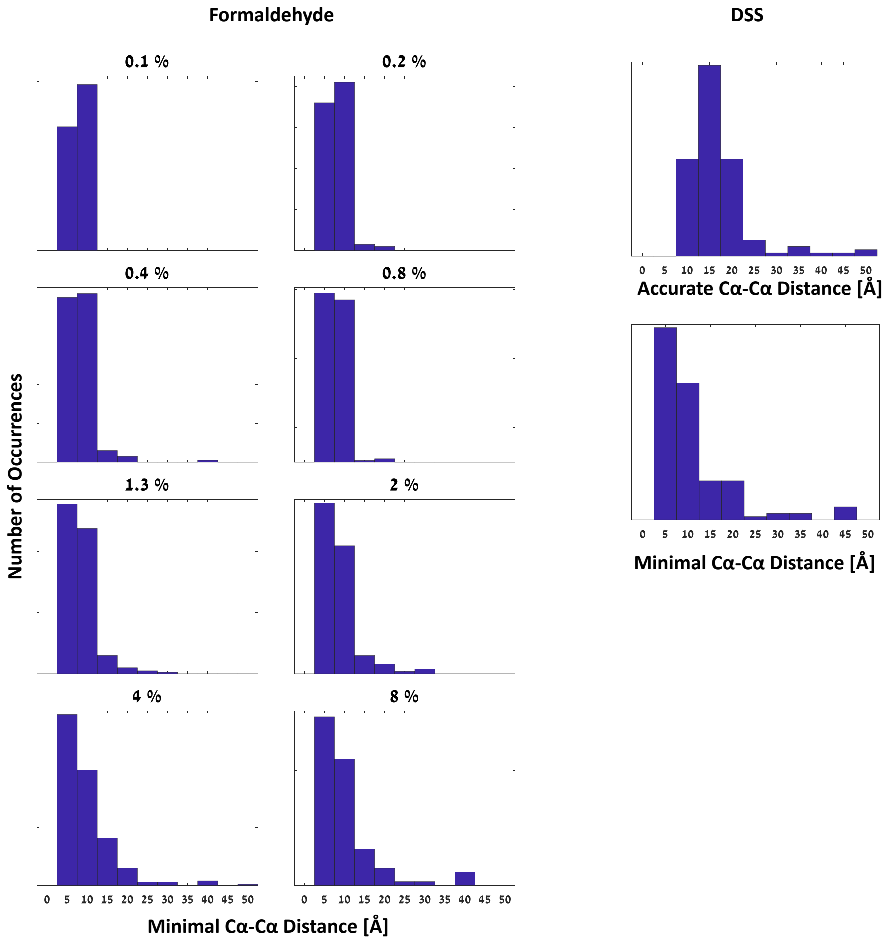

Supplementary Figure 6. Histograms of the distances between cross-linked peptides. The histograms are shown for eight formaldehyde concentrations **(left)** and for cross-linking with 3mM DSS (disuccinimidyl suberate) **(right)**. The cross-link distance was estimated as the minimal Cα-Cα distance found between the peptide pair on the crystallographic structure. For DSS cross-linking the linked sites are known without ambiguity and accurate Cα-Cα distances can be calculated **(right, top)**.

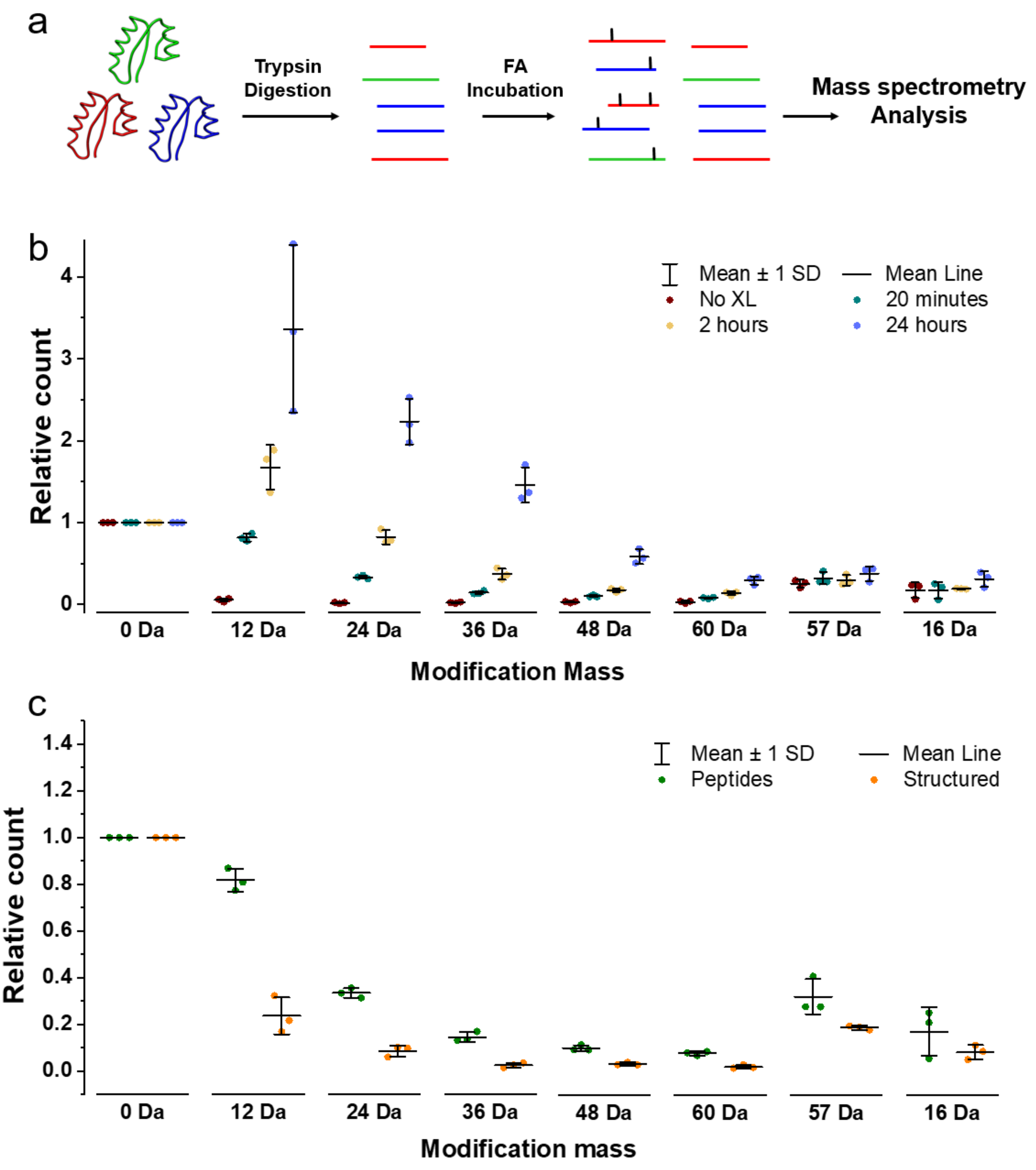

Supplementary Figure 7. Formaldehyde (FA) local modifications are fundamentally different from long-range cross-links. (a) A different experimental setup to study the effects of formaldehyde on peptides. Cross-links between two peptides could not be detected with this setup. **(b)** Search for FA modifications on linear peptides in mass spectrometry data from the setup described in (a). Each bar series is normalized by the count of non-modified peptides (0 Da modification mass). We searched for matches to multiple occurrences of the 12 Da reaction on the same peptide (24, 36, 48, and 60 Da). As a control, we also searched for peptides with off-target

alkylation (57 Da) and peptides with oxidized methionine residues (16 Da). For all FA incubation times, we see that peptides with a single 12 Da modification are the most frequent. FA concentration was 2%. **(c)** A similar search for modifications on linear peptides, but this time comparing the above setup (Panel a, Peptides) to the setup in Figure 1 (Structured). In both setups, the FA concertation was 2% and the incubation time was 20 minutes. Formaldehyde is less reactive towards structured proteins, forming fewer modifications. Means and standard deviations calculated across three independent experiments.
