# Supplementary material for: Mass spectrometry reveals the chemistry of formaldehyde cross-linking in structured proteins
Source: Nat Commun. 2020 Jun 19;11:3128. doi: 10.1038/s41467-020-16935-w (PMC7305180; doi:10.1038/s41467-020-16935-w)
Supplement: Supplementary file 3 — Description of Additional Supplementary Files [file 41467_2020_16935_MOESM3_ESM.pdf]

Description of Additional Supplementary Files

File name: Supplementary Data 1
Description: Cross-links identified in the MS data of 4% formaldehyde cross-linking of a three-protein mixture (Excel file).

File name: Supplementary Data 2
Description: Cross-links identified by Merox in the same MS data as Supplementary Data 1 (Excel file).

File name: Supplementary Data 3
Description: List of 559 cross-links identified from in situ XL-MS of human PC9 cells (Excel file).

File name: Supplementary Data 4
Description: Annotated MS/MS spectra of the cross-links listed in Table 1 (PDF file).
