# Supplementary material for: Mass spectrometry reveals the chemistry of formaldehyde cross-linking in structured proteins
Source: Nat Commun. 2020 Jun 19;11:3128. doi: 10.1038/s41467-020-16935-w (PMC7305180; doi:10.1038/s41467-020-16935-w)
Supplement: Supplementary file 7 — Supplementary Dataset 4 [file 41467_2020_16935_MOESM7_ESM.pdf]

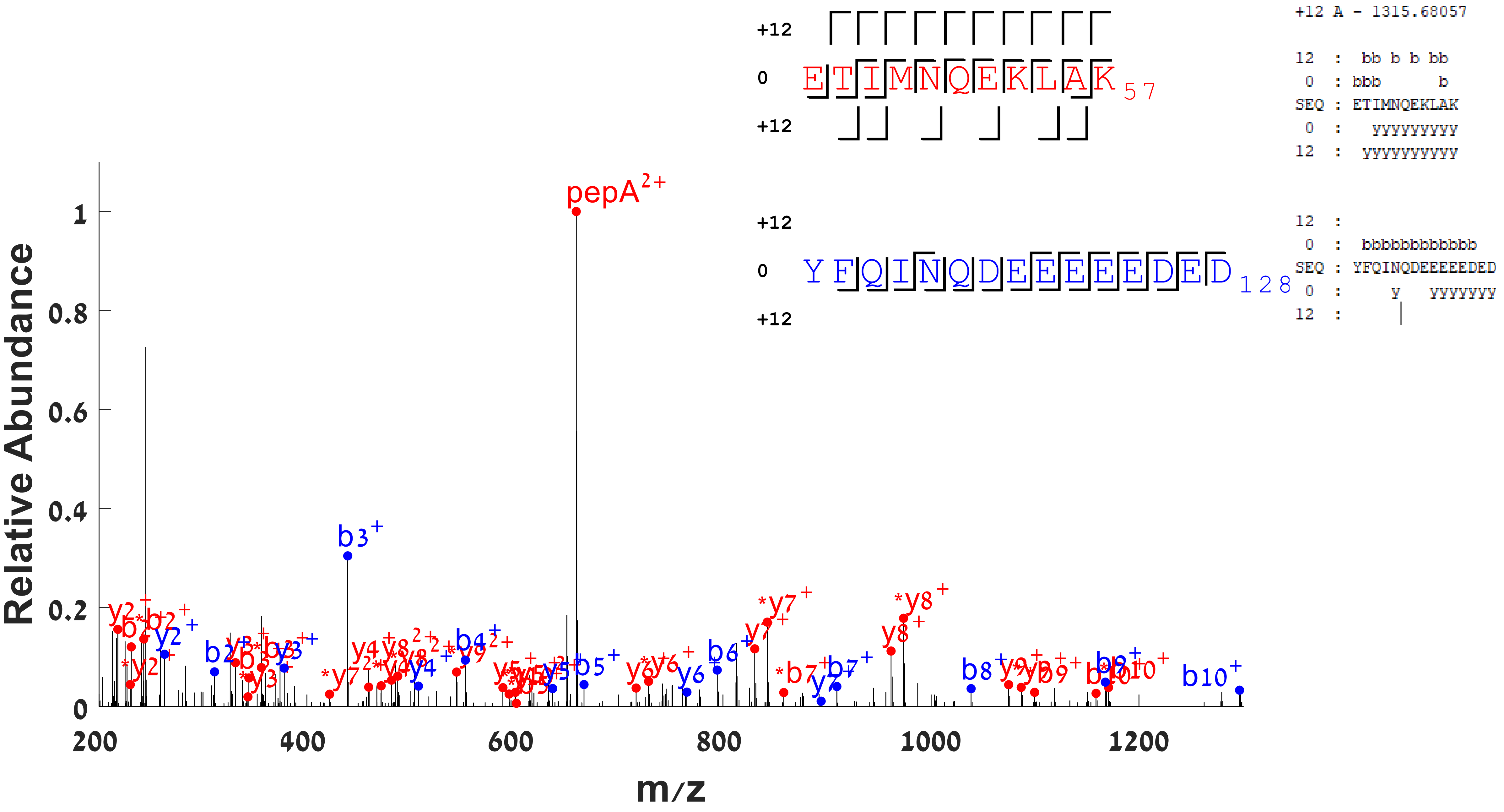

Row_22 BTF3_HUMAN RL22_HUMAN 47 114 ETIMNQEKLAK YFQINQDEEEEEDED-Cterm mass=12

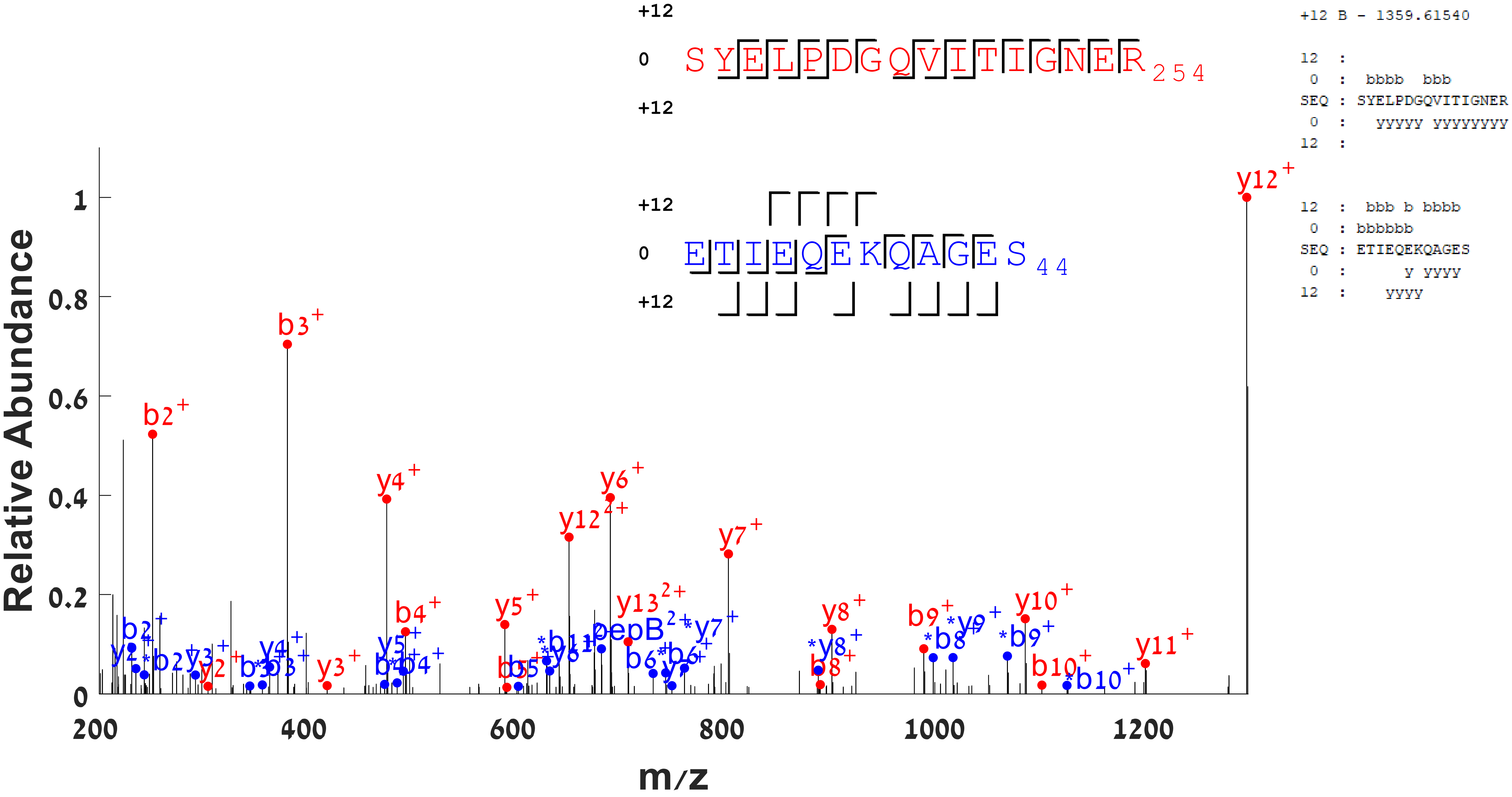

Row 26 ACTB_HUMAN TYB4_HUMAN 239 33 SYELPDGQVITIGNER ETIEQEKQAGES-Cterm mass=12

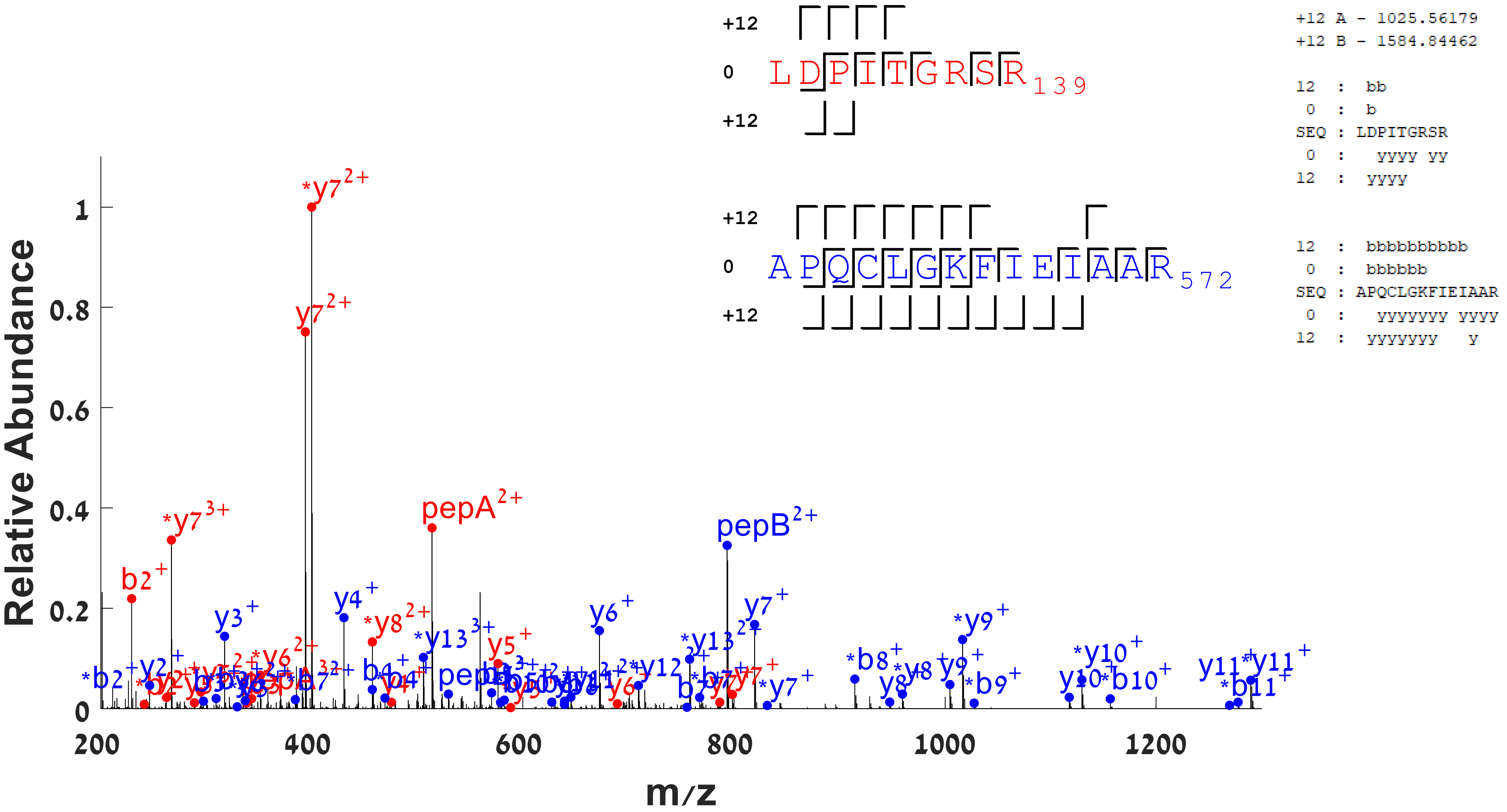

Row 28 HNRPD_HUMAN HNRPU_HUMAN 130 559 LDPITGRSR APQCLGKFIEIAAR mass=24

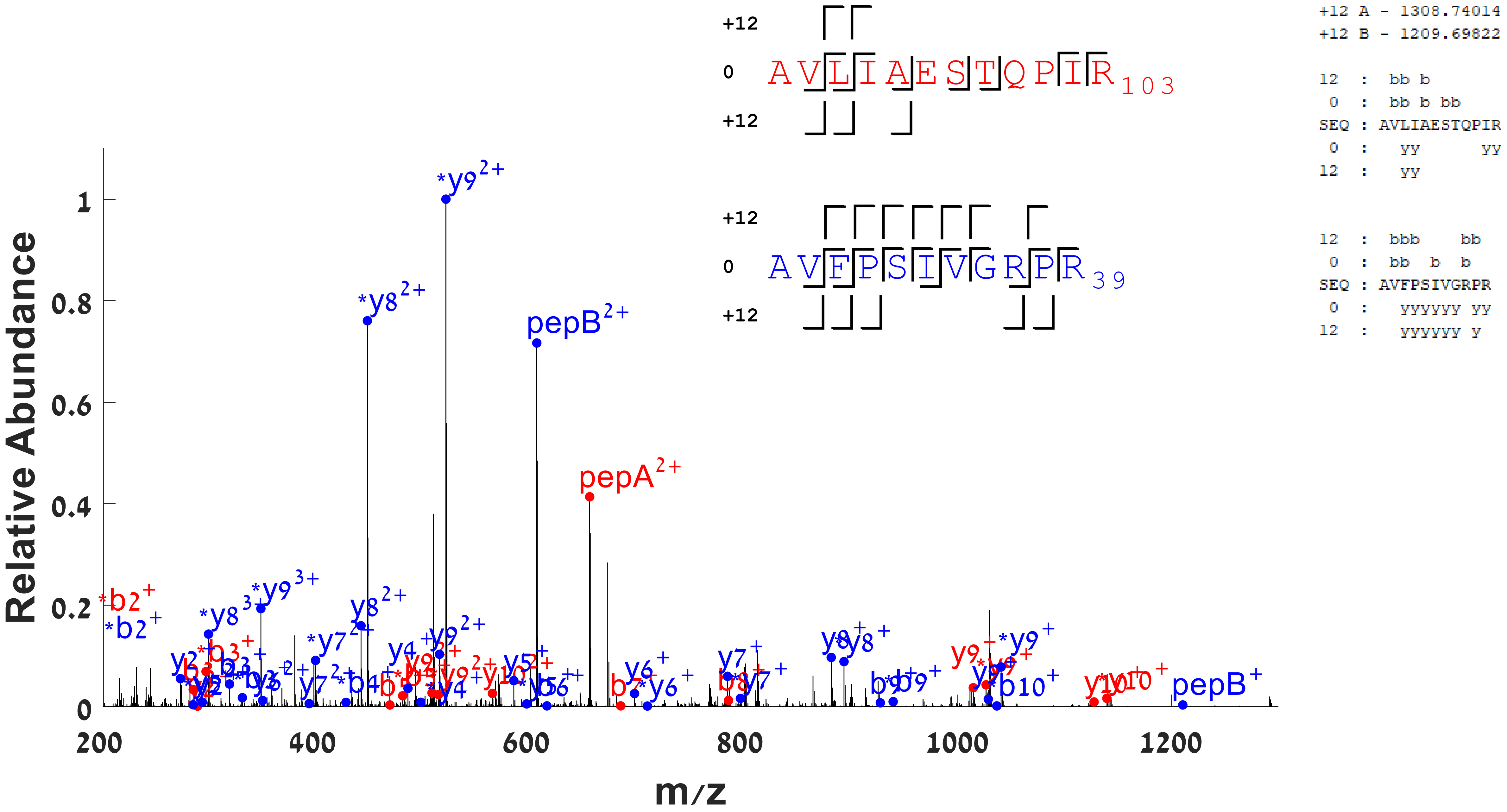

Row 29 P210L_HUMAN ACTB_HUMAN 92 29 AVLIAESTQPIR AVFPSIVGRPR mass=24

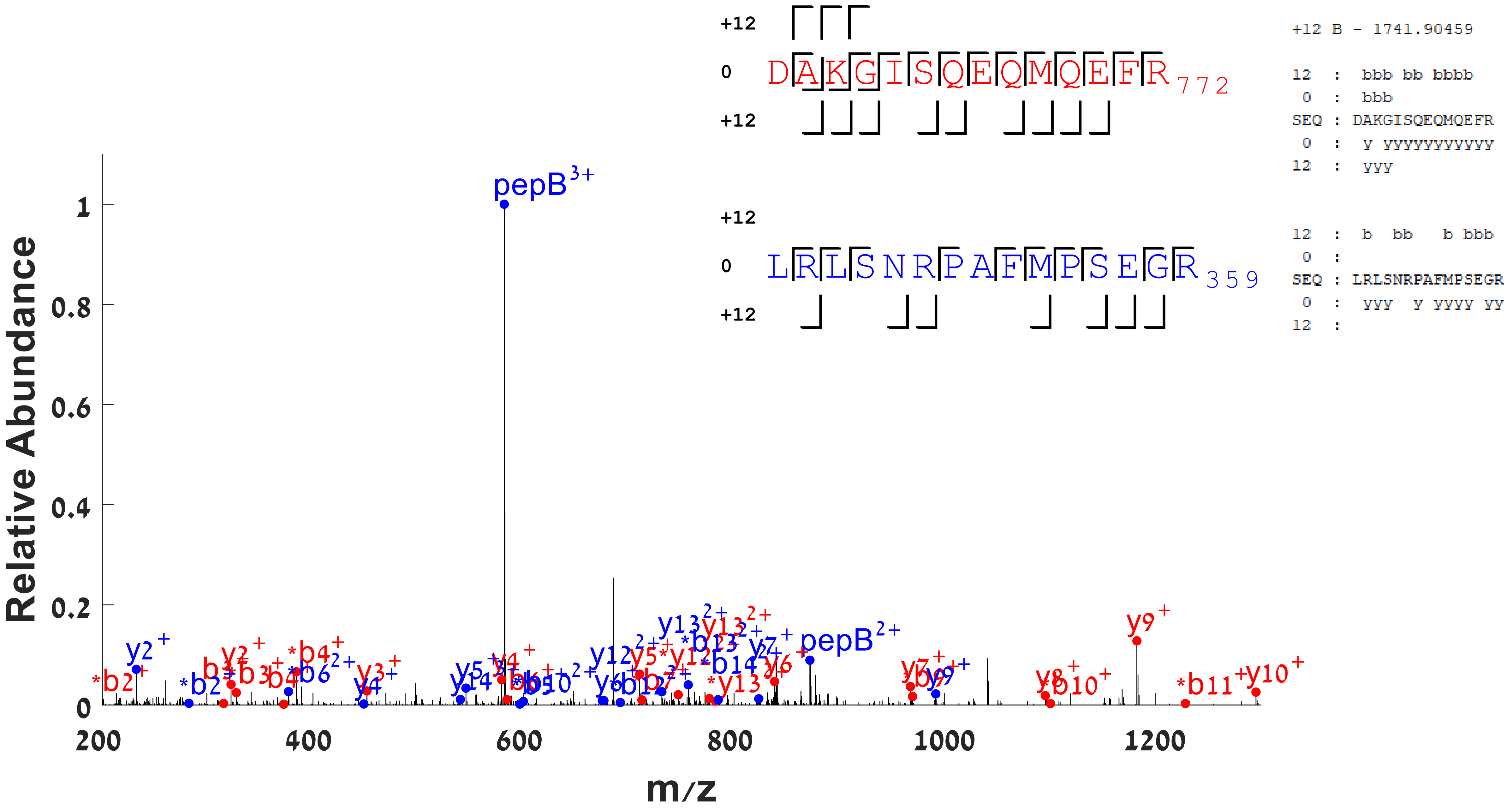

Row 31, ACTN4_HUMAN ACTN1_HUMAN 758 345 DAKGISQEQMQEFR LRLSNRPAFMPSEGR mass=24

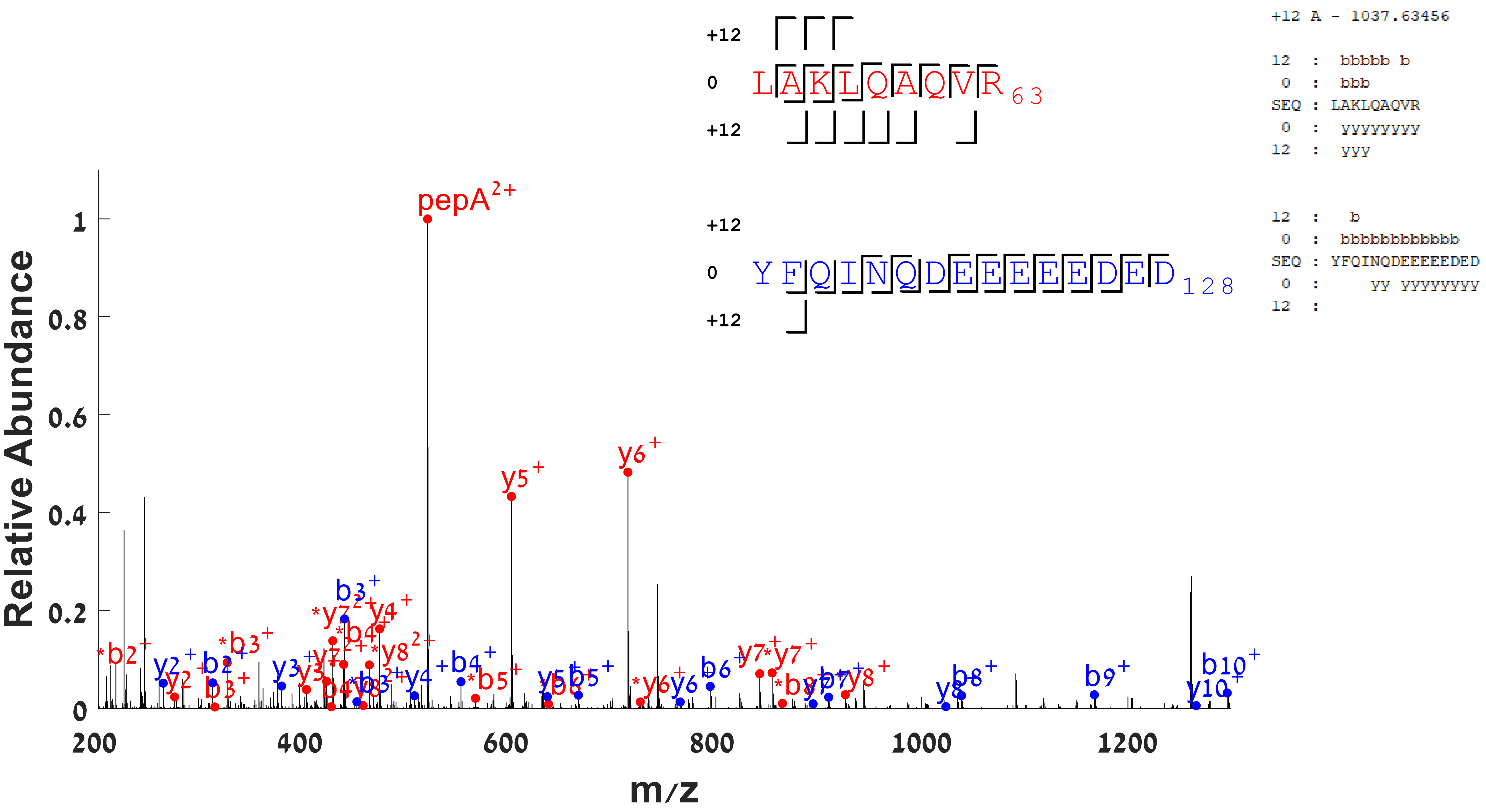

Row 32 BTF3_HUMAN RL22_HUMAN 55 114 LAKLQAQVR YFQINQDEEEEEDED-Cterm mass=12

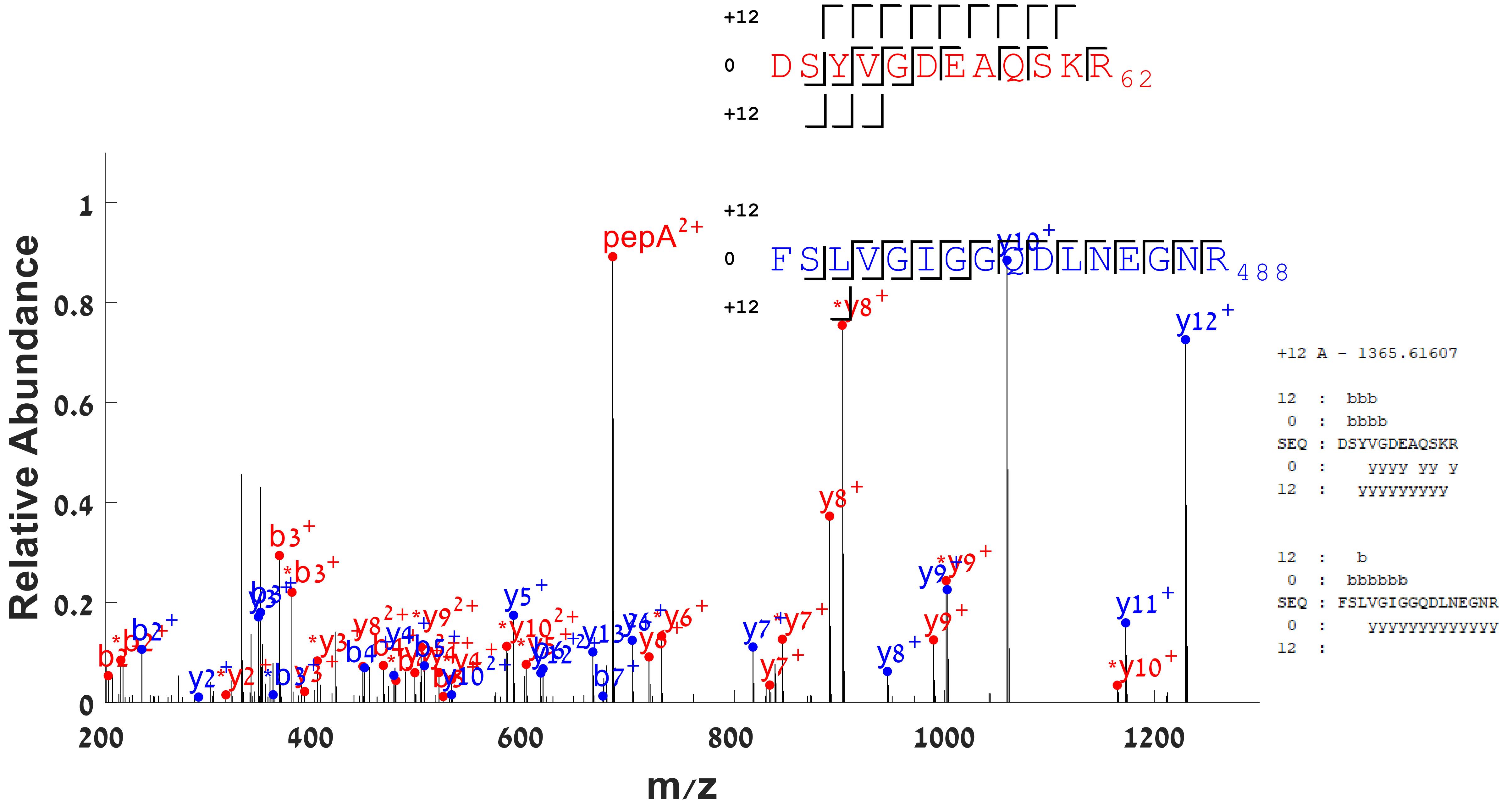

Row 40 ACTB_HUMAN PLSL_HUMAN 51 473 DSYVGDEAQSKR FSLVGIGGQDLNEGNR mass=12

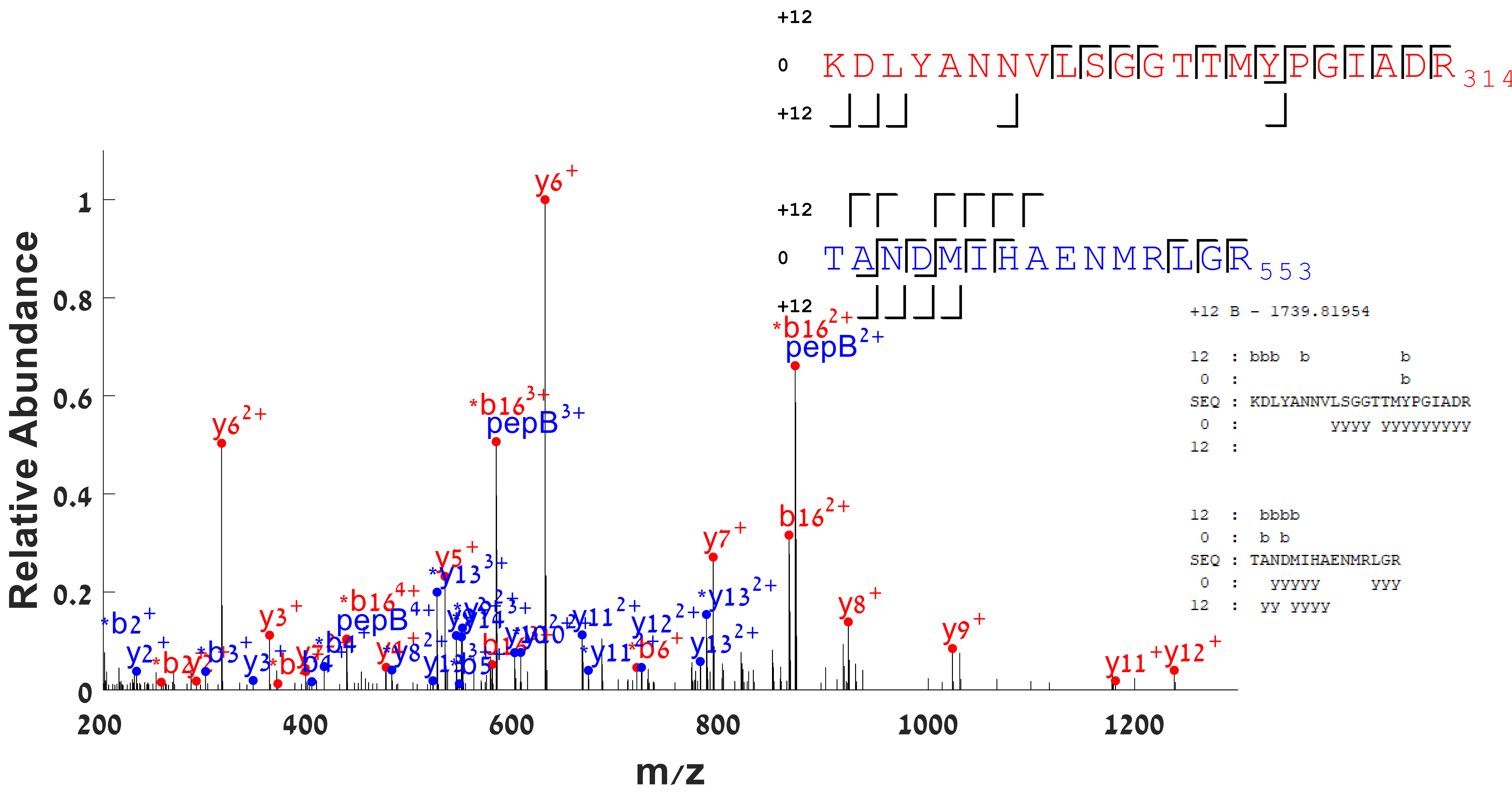

Row 41 ACTC_HUMAN MOES_HUMAN 293 539 KDLYANNVLSGGTTMYPGIADR TANDMIHAENMRLGR mass=12

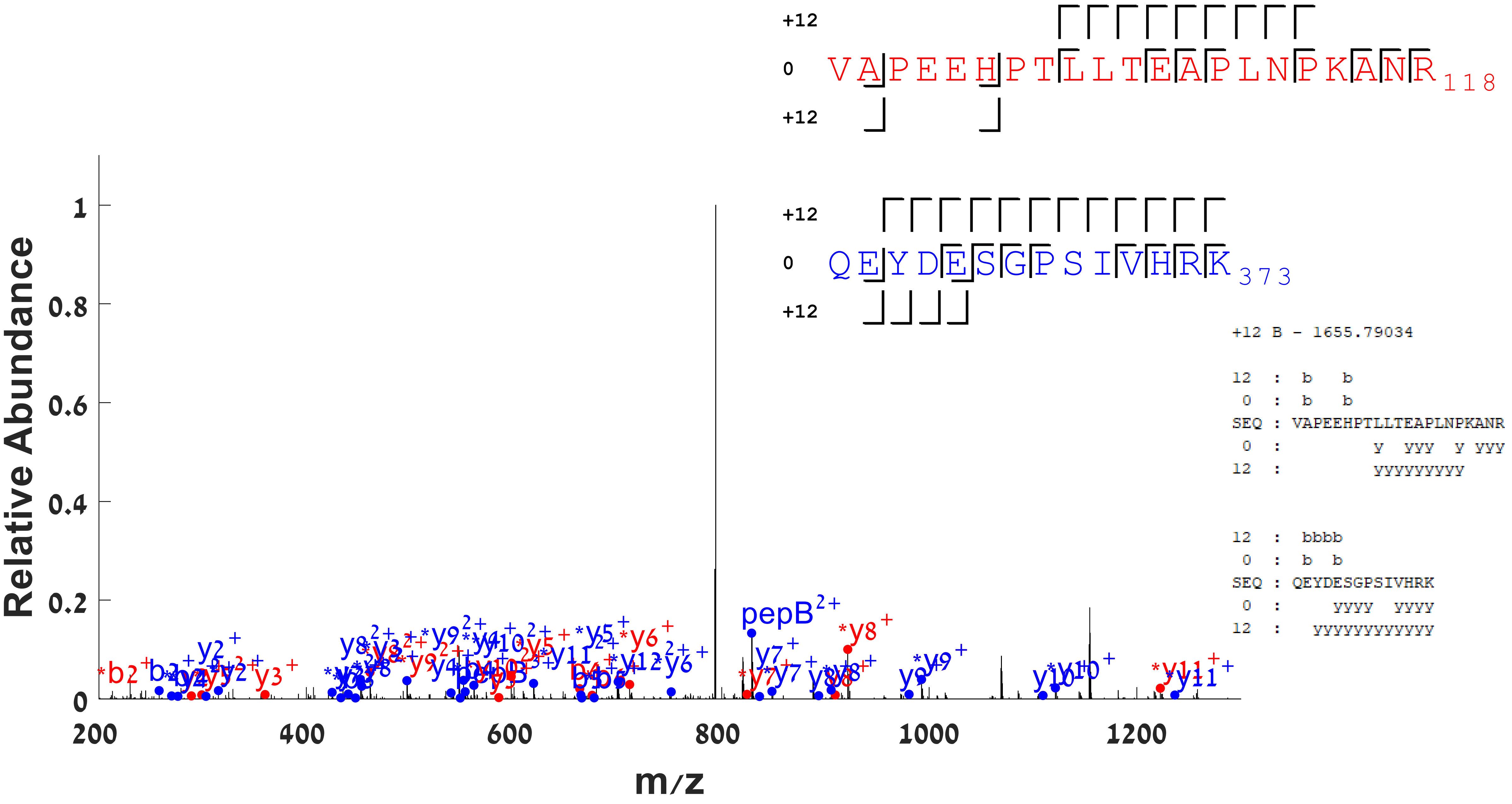

Row 47 ACTC_HUMAN ACTB_HUMAN 98 360 VAPEEHPTLLTEAPLNPKANR QEYDESGPSIVHRK mass=24

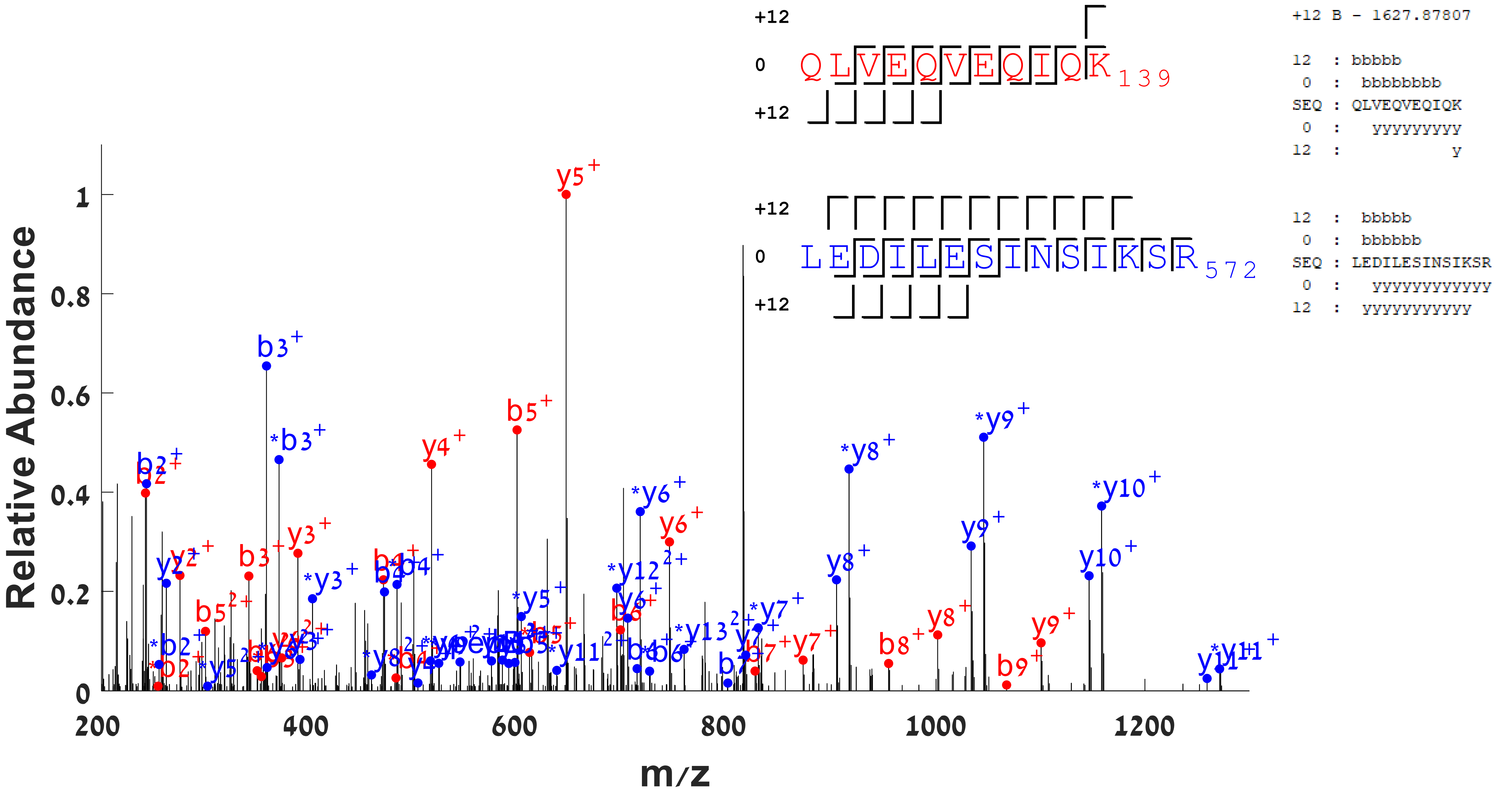

Row 60 TMED9_HUMAN TMED5_HUMAN 170 153 QLVEQVEQIQK LEDILESINSIKSR mass=12

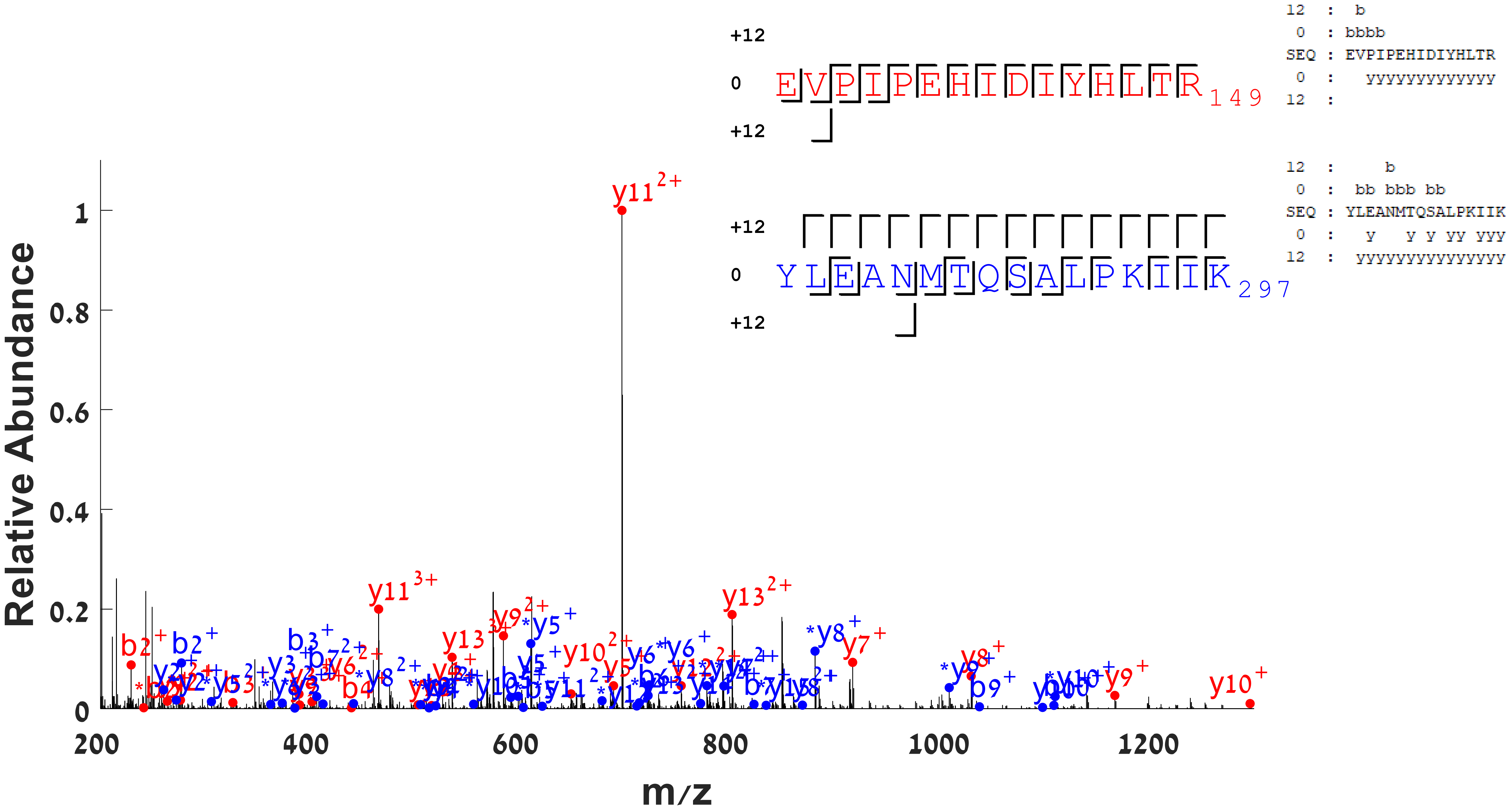

Row 61 ABCF2_HUMAN OLA1_HUMAN 135 282 EVPIPEHIDIYHLTR YLEANMTQSALPKIIK mass=12

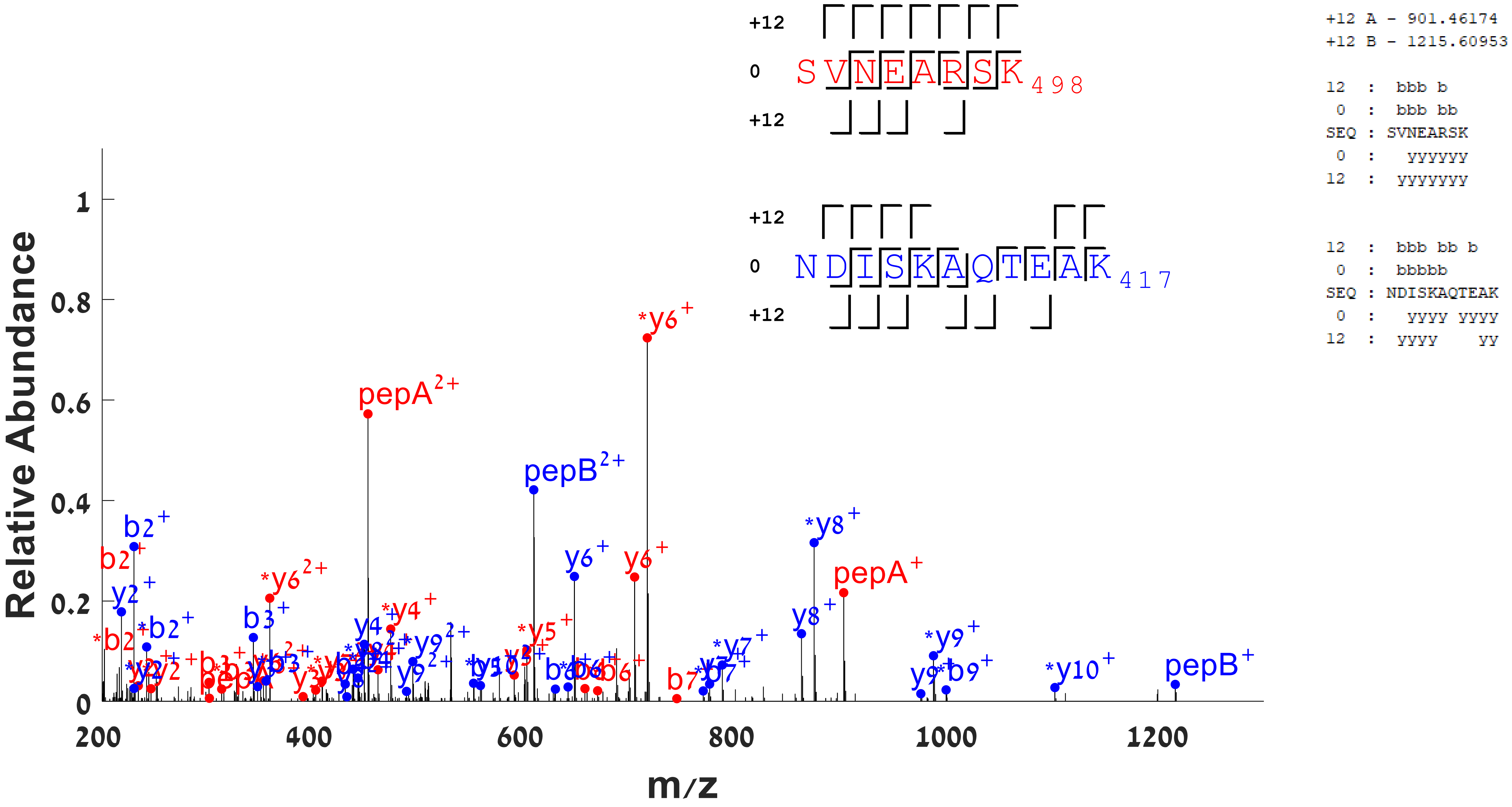

Row 65 SMC4_HUMAN SMC2_HUMAN 491 407 SVNEARSK NDISKAQTEAK mass=24

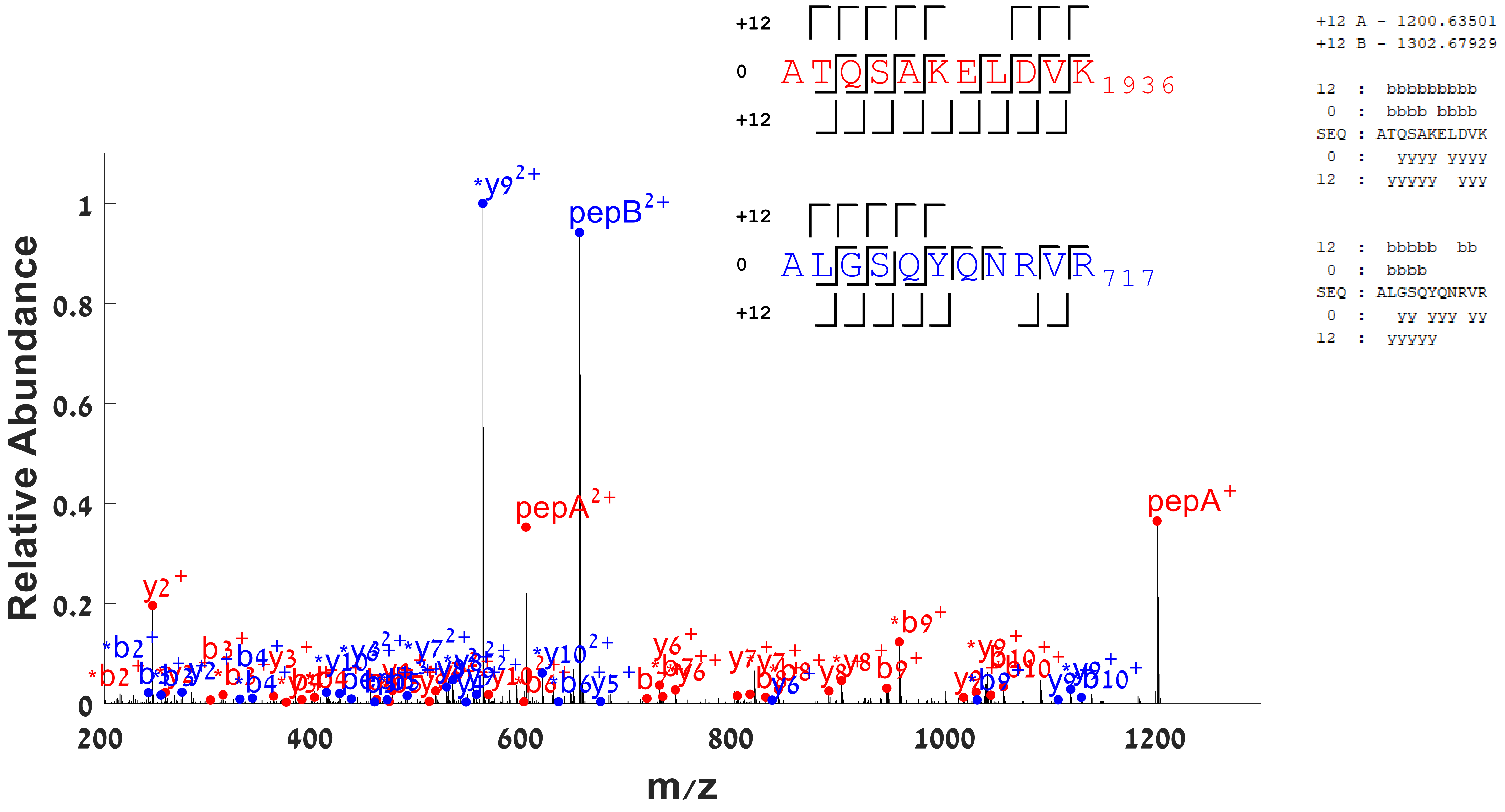

Row 67 LAMA3_HUMAN LAMC2_HUMAN 1926 707 ATQSAKELDVK ALGSQYQNRVR mass=24

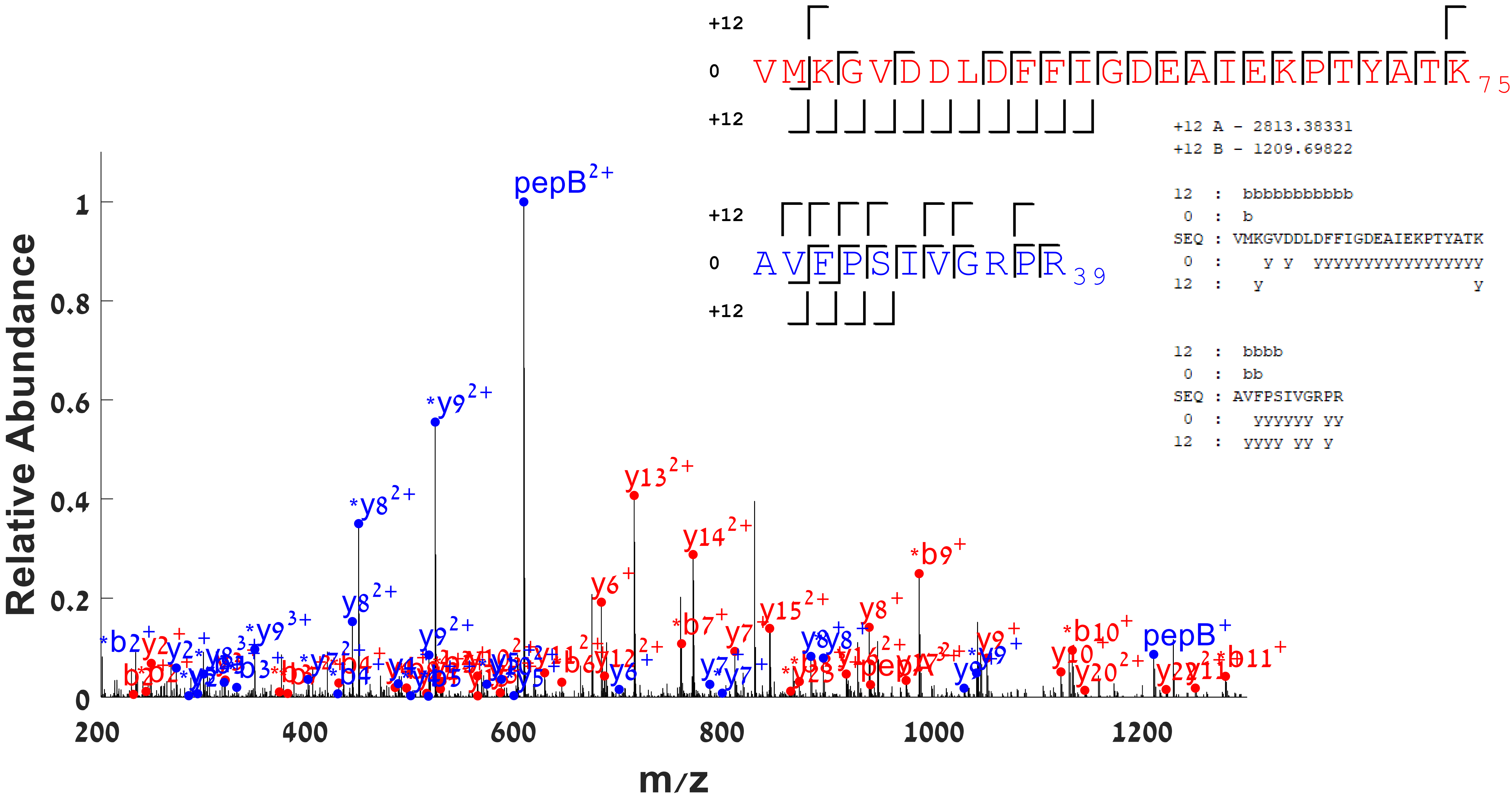

Row 74 ARP3_HUMAN ACTB_HUMAN 51 29 VMKGVDDLDFFIGDEAIEKPTYATK AVFPSIVGRPR mass=24

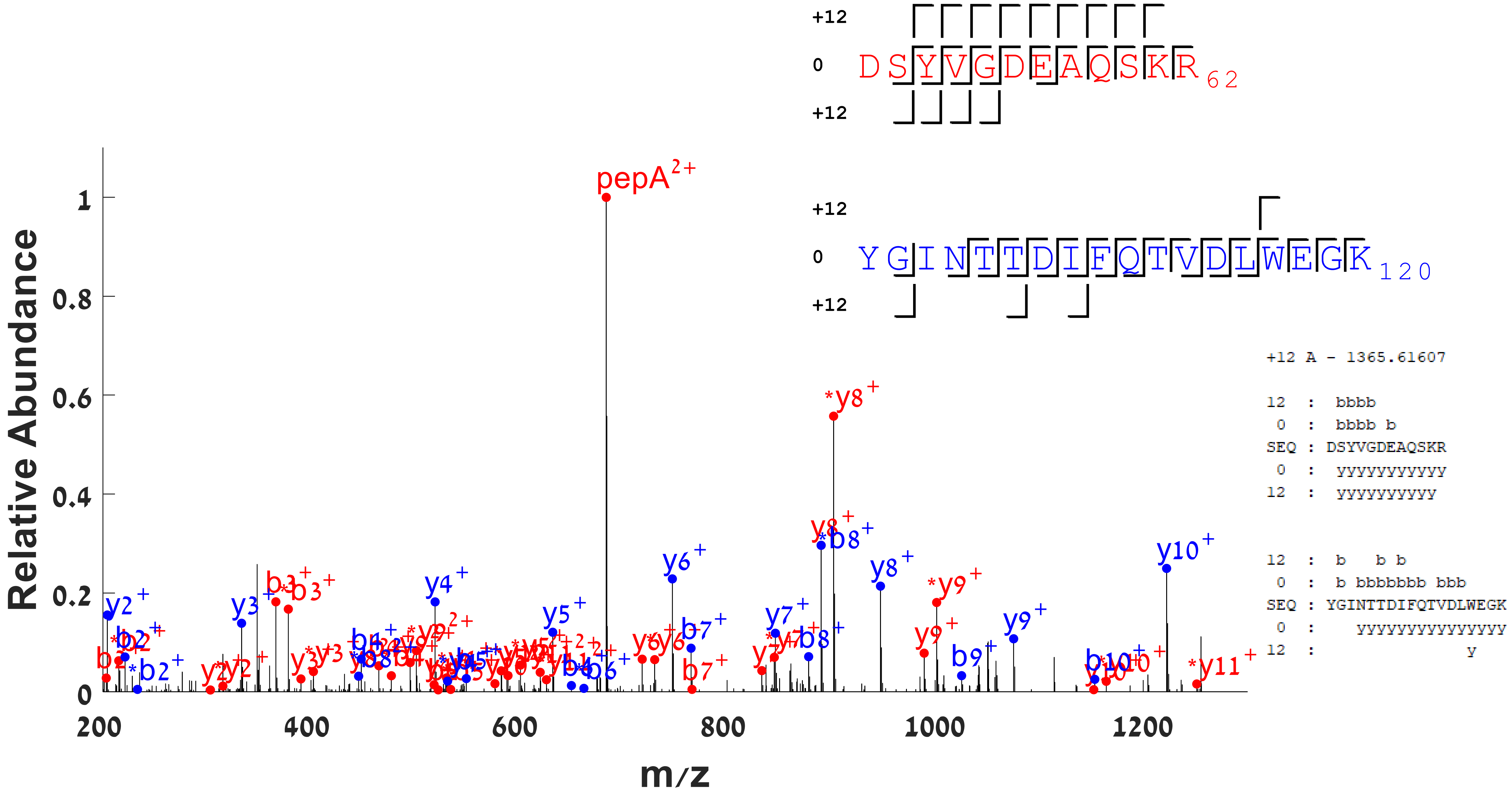

Row 76 ACTB_HUMAN TAGL2_HUMAN 51 103 DSYVGDEAQSKR YGINTTDIFQTVDLWEGK mass=12

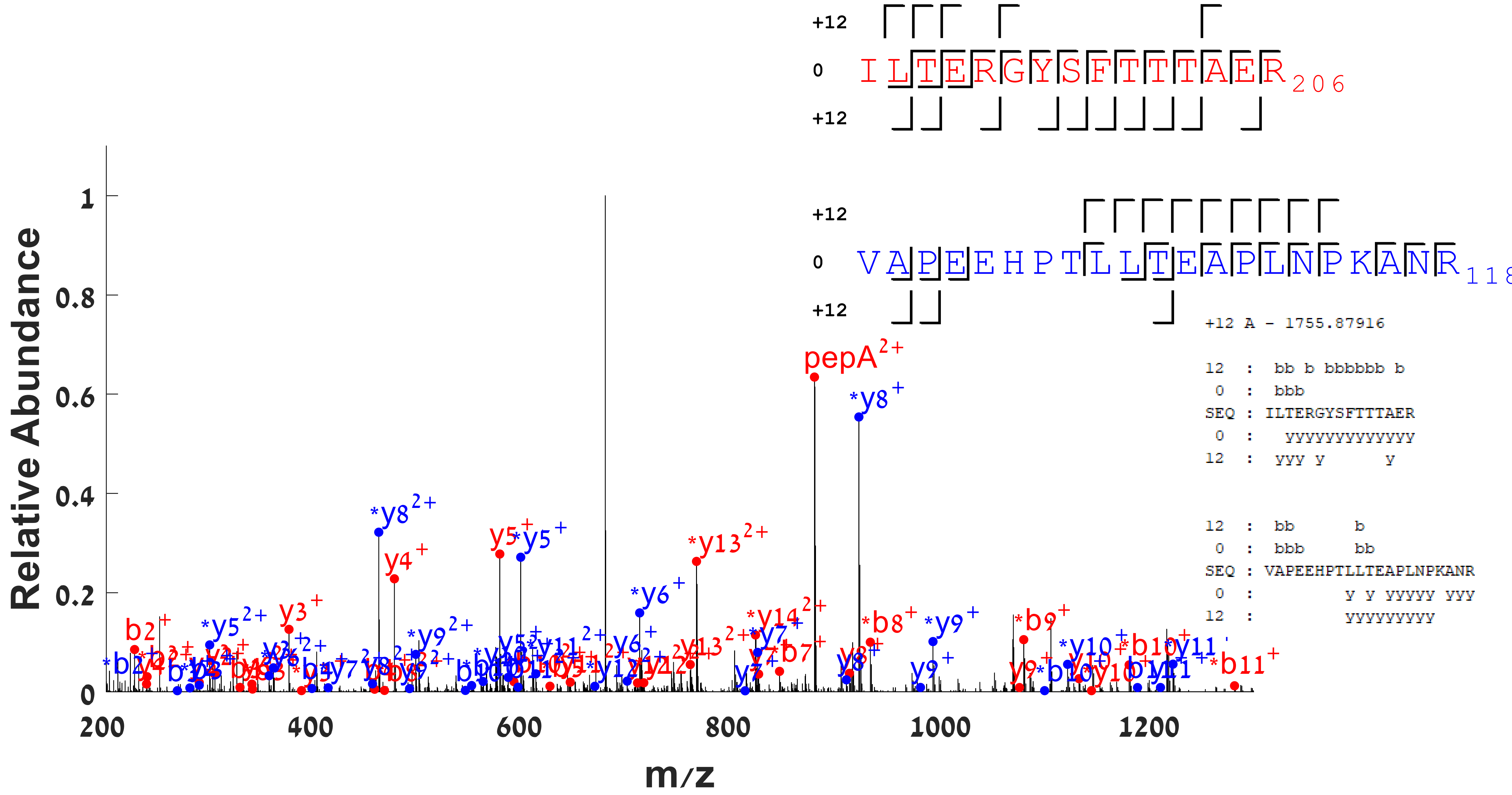

Row 78 ACTB_HUMAN ACTC_HUMAN 192 98 ILTERGYSFTTTAER VAPEEHPTLLTEAPLNPKANR mass=24
