# Supplementary material for: Mass spectrometry reveals the chemistry of formaldehyde cross-linking in structured proteins
Source: Nat Commun. 2020 Jun 19;11:3128. doi: 10.1038/s41467-020-16935-w (PMC7305180; doi:10.1038/s41467-020-16935-w)
Supplement: Supplementary file 2 — Peer Review File [file 41467_2020_16935_MOESM2_ESM.pdf]

Reviewer #1 (Remarks to the Author):

Review of NCOMMS-20-09835

I am quite positive about this paper. The issue the authors tackle, of no one ever finding "the smoking gun" of formaldehyde crosslinking, in the form of the anticipated modified peptides or crosslinked peptide products, is one that has vexed me and vexed colleagues. Formaldehyde crosslinking is really used a LOT! FFPE tissue samples is one huge domain. In addition ChIP, ChIP-chip, ChIP-seq, Hi-C, and many other widely used techniques all rely upon formaldehyde crosslinking. It seemed obvious that if it crosslinks things, one should be able to see the crosslinked products by mass spec, yet somehow, in spite of so much work, those products have not been visible. It is almost embarrassing for the chemistry enterprise as a whole, to have such an important and widespread chemistry, using a molecule with only 4 atoms, to somehow have a mysterious and unknown chemical behavior that behaves in a way we do not understand and defies predictions.

So, the authors have done a great job here, of using simple reasoning and straightforward methods to ferret out the origin of the mystery, and they provide both a simple and straightforward mechanism and an approach that will allow them and others to see the reaction products henceforth. It is a great service to the field, removing a cloak of darkness that has hung over an important and very widely used chemistry.

Aside this global positivity, I have a few minor comments, mostly small grammatical items..

- Abstract line 29 IO suggest "The mass spectrometry data…" rather than "Our mass spectrometry data…"
- Line 31. I find the term "surface modifications" a bit odd. I don't feel like the chemistry is modifying a surface, it is modifying amino acids on a protein which are presumably exposed to solvent… I suggest "amino acid modifications" ?
- Intro line 64 delete "a" before "commonly"
- Line 124 no need for the hyphen in "undergoing"
- Line 152 delete "is" before "dominates"
- Line 175 insert "on" before "its efficiency"
- Line 208 "restrains" is missssspelled (sic)
- Line 219 insert the word "comprise" before "1.6%"
- Sentence line 238-239 is unclear to me… I don't follow the logic of "The mild reactivity…suggests that they underlie …toxic effects…" Need to spell out the logic of this thought I'm not following.
- Line 301 "Fractions" not "Fraction"

In Figure 5 there is an "e" missing panel A "Formaldehyd "

Also I am checking to see if the authors are confident in what they are saying in Panel B – "a nucleophilic attack by the imine on a nearby lysine sidechain" - this indicates that the imine is more nucleophilic than the lysine primary amine, which I find surprising as such primary amines are generally considered to be strong nucleophiles. A bit of googling turned up this "There is therefore less + charge at the imine carbon atom which makes it less electrophilic; thus whilst imines do react with nucleophiles in a similar fashion to carbonyl groups, they tend to require strong nucleophiles and more forcing reaction conditions. "

Supporting the general idea that in this reaction the imine is the electrophile and the amine the nucleophile. If my chemistry building was not closed due to the Covid-19 pandemic I have a dozen colleagues who could answer this question for me in a millisecond.. however I am on my own, and so I just suggest the authors carefully consider their statement, it would be a shame to have such a nice paper include a possibly incorrect statement, especially in the most important figure that contains the major "take-home" message of the paper.

A final point to consider relates to reversibility.

The mechanism in Fig. 5 should show double arrows for all reactions to indicate reversibility, and then this supposition of reversibility should be mentioned/discussed in the text somewhere. All indications in the world are that formaldehyde is a reversible crosslinker, and therefore all steps of the mechanism are quite likely reversible. And there is no leaving group, just a concerted reaction that rearranges electrons, which is highly likely to be reversible. And the MS2 data presented provides strong evidence of reversibility.

Reviewer #2 (Remarks to the Author):

The manuscript from Tayri-Wilk, Slavin, and Zamel et al re-examines the characterization of formaldehyde crosslinks by mass spectrometry. The accepted mechanism, which is decades old, is that protein amines are crosslinked via a methylene bridge after initial Schiff base formation. The mechanism supposed here,
is that two proximal Schiff bases undergo a cycloadditon to form a 1,3-diazetidine linkage. This is a highly ring strained 4-membered ring with two carbon atoms each bonded to two heteroatoms, and I find this structure implausible.

However, overall the data presented here are actually fairly compelling. The idea of mass modification searching is common to look for linear modifications, but applying this strategy to scanning the crosslinker bridge mass is novel. The high-prevalence of +24 related modifications in the mod-histogram is a solid piece of evidence, and this is supported by the evidence that after gas-phase collisional dissociation of the
crosslinked peptides, the dissociation products are consistent with two linear peptide each modified by +12. Furthermore, the mappable crosslinks are consistent with known crystallographic structure and only form on structured proteins.

FA is a highly attractive reagent to use for large-scale crosslinking studies due to its membrane permeability and long history of applications in a variety of cell and tissue fixation applications. The feasibility of FA-CLMS analysis where FA can be searched as a gas-phase labile linker analogous to DSSO is a rather tasty idea.

As mentioned, I do have trouble swallowing the cyclic intermediate proposed. However, the manuscript should focus on the practical aspects of analyzing CLMS data using this approach, rather than getting bogged down in reaction mechanism. My suggestions below are oriented towards working out more basic aspects of this chemistry for the purpose of improving the robustness of the applications.

1) The inability to find FA crosslinks is typically attributed to instability of the methylene bridged intermediates. The authors note that they keep their experimental preps below 40C to prevent decomposition. The addition of some experiments that explicitly characterized the chemical stability of FA crosslinks would be highly
helpful to defining the general utility of this method. Particularly with respect to pH and temperature as these sample preparation and enrichment strategies used in proteomics, might for instance require high pH fractionation.

2) The authors strategy for searching complex databases utilized a +24 Da variable modification with no residue specificity. This is inefficient and also likely to increase the error rate of this type of search. More experiments to define the amino acid specificity of FA crosslinking would be both useful and help with clarifying the mechanism. There are a number of papers by Kast group and others that have probed this extensively with linear peptides. I believe the Kast paper (ref 10) shows that at mild reaction conditions and short times, primarily Lys and Arg are modified and that other amino acids are

only FA modified after days of reaction. Perusing the supplemental spectra here, there are cases where there are no K residues in the peptides and the pattern of +12 containing product ions (called B- and Y-ion here, see comment below) imply a modification on residues with no modifiable heteroatoms (I, L, V G, etc.). (eg P210L_HUMANACTB_HUMAN9229AVLIAESTQPIRAVFPSIVGRPR). I recommend searching the standard protein data with different residue specificity to try to establish the reactivity profile more clearly.

3) The methods section does not mention the collision parameters for the HCD. Definitely add this, but also a study optimizing this should be added. In particular, is it possible at low energy to preferentially cleave the crosslink but leave the peptides largely intact for MS3 targeting. If the author have access to an ETD enabled instrument, I would also be interested in seeing whether the FA bridges are dissociated by electron transfer.

minor -

A) The authors mention that there is no direct evidence for observation of methylene bridged crosslinks. While I don't disagree with their general conclusions, (ie model peptide based reactions are a poor way to study this), I don't strictly speaking think this is true. For instance, ref 5, finds reaction between model peptide and glycine that are mediated by FA. Table I in that reference shows that a mass difference of +87 Da (eg Gly + 12Da) is the most common adduct. However, same table also finds +99 Da adducts which they attribute to multiple methylene bridges, but are also consistent with the mechanism here.

B) Their search software annotates product ions that have a +12 Da modification as B- and Y- ions (upper case). I would avoid this, since this nomenclature is already well established for Glycopeptide fragmentation.

C) For the mass modification scans - are these scanned as integer Dalton values? General practice is to model the average peptide distribution ("averagine") and add a mass defect based on this.

D) Authors should probably formally separate out searching for mis-annotated mono-isotopic masses and the crosslink search in the search engine. Searching for +24, +25, +26 is fairly awkward and could be handled by a neutral loss search for 1 or 2-Da.

REVIEWER COMMENTS

Reviewer #1 (Remarks to the Author):

Review of NCOMMS-20-09835

I am quite positive about this paper. The issue the authors tackle, of no one ever finding “the smoking gun” of formaldehyde crosslinking, in the form of the anticipated modified peptides or crosslinked peptide products, is one that has vexed me and vexed colleagues. Formaldehyde crosslinking is really used a LOT! FFPE tissue samples is one huge domain. In addition ChIP, ChIP-chip, ChIP-seq, Hi-C, and many other widely used techniques all rely upon formaldehyde crosslinking. It seemed obvious that if it crosslinks things, one should be able to see the crosslinked products by mass spec, yet somehow, in spite of so much work, those products have not been visible. It is almost embarrassing for the chemistry enterprise as a whole, to have such an important and widespread chemistry, using a molecule with only 4 atoms, to somehow have a mysterious and unknown chemical behavior that behaves in a way we do not understand and defies predictions.

So, the authors have done a great job here, of using simple reasoning and straightforward methods to ferret out the origin of the mystery, and they provide both a simple and straightforward mechanism and an approach that will allow them and others to see the reaction products henceforth. It is a great service to the field, removing a cloak of darkness that has hung over an important and very widely used chemistry.

We thank the reviewer for these encouraging comments.

Aside this global positivity, I have a few minor comments, mostly small grammatical items.

- Abstract line 29 IO suggest “The mass spectrometry data…” rather than “Our mass spectrometry data…”
- Line 31. I find the term “surface modifications” a bit odd. I don’t feel like the chemistry is modifying a surface, it is modifying amino acids on a protein which are presumably exposed to solvent… I suggest “amino acid modifications” ?
- Intro line 64 delete “a” before “commonly”
- Line 124 no need for the hyphen in “undergoing”
- Line 152 delete “is” before “dominates”
- Line 175 insert “on” before “its efficiency”
- Line 208 “restrains” is missssspelled (sic)
- Line 219 insert the word “comprise” before “1.6%”
- Sentence line 238-239 is unclear to me… I don’t follow the logic of “The mild reactivity…suggests that they underlie …toxic effects…” Need to spell out the

logic of this thought I’m not following. – The entire sentence was removed.
- Line 301 “Fractions” not “Fraction”
In Figure 5 there is an “e” missing panel A “Formaldehyd “

All items corrected. We thank the reviewer for carefully spotting these grammatical errors.

Also I am checking to see if the authors are confident in what they are saying in Panel B – “a nucleophilic attack by the imine on a nearby lysine sidechain” - this indicates that the imine is more nucleophilic than the lysine primary amine, which I find surprising as such primary amines are generally considered to be strong nucleophiles. A bit of googling turned up this “There is therefore less + charge at the imine carbon atom which makes it less electrophilic; thus whilst imines do react with nucleophiles in a similar fashion to carbonyl groups, they tend to require strong nucleophiles and more forcing reaction conditions. “
Supporting the general idea that in this reaction the imine is the electrophile and the amine the nucleophile. If my chemistry building was not closed due to the Covid-19 pandemic I have a dozen colleagues who could answer this question for me in a millisecond.. however I am on my own, and so I just suggest the authors carefully consider their statement, it would be a shame to have such a nice paper include a possibly incorrect statement, especially in the most important figure that contains the major “take-home” message of the paper.

We completely agree with the reviewer, and acknowledge this mistake in the figure. The sentence was changed to “Nucleophilic attack by a nearby lysine sidechain”

A final point to consider relates to reversibility.

The mechanism in Fig. 5 should show double arrows for all reactions to indicate reversibility, and then this supposition of reversibility should be mentioned/discussed in the text somewhere. All indications in the world are that formaldehyde is a reversible crosslinker, and therefore all steps of the mechanism are quite likely reversible. And there is no leaving group, just a concerted reaction that rearranges electrons, which is highly likely to be reversible. And the MS2 data presented provides strong evidence of reversibility.

The reactions in Fig. 5 are now shown with double arrows. The following sentences were added to the Discussion (line 259): “The dimerization is consistent with the known reversibility of formaldehyde cross-linking, which implies that all steps of the mechanism are reversible. In particular, the MS/MS spectra clearly demonstrate the full reversal of the last dimerization step by the introduction of mild collision energy.”

Reviewer #2 (Remarks to the Author):

The manuscript from Tayri-Wilk, Slavin, and Zamel et al re-examines the characterization of formaldehyde crosslinks by mass spectrometry. The accepted mechanism, which is decades old, is that protein amines are crosslinked via a methylene bridge after initial Schiff base formation. The mechanism supposed here, is that two proximal Schiff bases undergo a cycloadditon to form a 1,3-diazetidine linkage. This is a highly ring strained 4-membered ring with two carbon atoms each bonded to two heteroatoms, and I find this structure implausible.

However, overall the data presented here are actually fairly compelling. The idea of mass modification searching is common to look for linear modifications, but applying this strategy to scanning the crosslinker bridge mass is novel. The high-prevalence of +24 related modifications in the mod-histogram is a solid piece of evidence, and this is supported by the evidence that after gas-phase collisional dissociation of the crosslinked peptides, the dissociation products are consistent with two linear peptide each modified by +12. Furthermore, the mappable crosslinks are consistent with known crystallographic structure and only form on structured proteins.

FA is a highly attractive reagent to use for large-scale crosslinking studies due to its membrane permeability and long history of applications in a variety of cell and tissue fixation applications. The feasibility of FA-CLMS analysis where FA can be searched as a gas-phase labile linker analogous to DSSO is a rather tasty idea.

As mentioned, I do have trouble swallowing the cyclic intermediate proposed. However, the manuscript should focus on the practical aspects of analyzing CLMS data using this approach, rather than getting bogged down in reaction mechanism. My suggestions below are oriented towards working out more basic aspects of this chemistry for the purpose of improving the robustness of the applications.

We completely agree that the current data cannot determine conclusively what is the exact chemical structure of the linkage. We therefore rephrase our conclusions in a more careful manner:
- The explicit chemical structure previously drawn in Fig. 5C was removed.
- The following text was added to the Discussion (line 255): “The current data cannot conclusively determine what is the chemical structure of the linkage site. One possibility is that the two imines undergo cycloaddition to form a 1,3-diazetidine linkage. Such a strained ring structure would be consistent with the tendency of the link to break completely under HCD fragmentation. Nonetheless, other chemical structures are equally possible and efforts to better characterize the linkage site by NMR are ongoing.”

1) The inability to find FA crosslinks is typically attributed to instability of the

methylene bridged intermediates. The authors note that they keep their experimental preps below 40C to prevent decomposition. The addition of some experiments that explicitly characterized the chemical stability of FA crosslinks would be highly helpful to defining the general utility of this method. Particularly with respect to pH and temperature as these sample preparation and enrichment strategies used in proteomics, might for instance require high pH fractionation.

With the COVID19-related interruptions it is uncertain when we can resume the regular lab work. We note that the temperatures (RT to 37°C) and pH (physiological 7.4) currently used in the manuscript were chosen primarily to be compatible with the conditions of *in situ* cross-linking. We also note that the 24Da reaction is compatible in our hands with SCX enrichment and MS buffers. Hence, it is stable down to pH values of 2-3.

2) The authors strategy for searching complex databases utilized a +24 Da variable modification with no residue specificity. This is inefficient and also likely to increase the error rate of this type of search. More experiments to define the amino acid specificity of FA crosslinking would be both useful and help with clarifying the mechanism. There are a number of papers by Kast group and others that have probed this extensively with linear peptides. I believe the Kast paper (ref 10) shows that at mild reaction conditions and short times, primarily Lys and Arg are modified and that other amino acids are only FA modified after days of reaction. Perusing the supplemental spectra here, there are cases where there are no K residues in the peptides and the pattern of +12 containing product ions (called B- and Y-ion here, see comment below) imply a modification on residues with no modifiable heteroatoms (I, L, V G, etc.). (eg P210L_HUMANACTB_HUMAN9229AVLIAESTQPIRAVFPSIVGRPR). I recommend searching the standard protein data with different residue specificity to try to establish the reactivity profile more clearly.

We followed this good suggestion with an analysis of the most likely link sites on the cross-linked peptides. We found that the majority of formaldehyde cross-links occur between Lys or Arg residues - consistent with previous reports by the groups of Kast, Jiskoot, and others. However, a significant fraction of the cross-links (almost a third) involves other amino acids (Asn, His, Asp, Tyr, and Gln). We therefore think that limiting the reactivity profile in the search only to Lys and Arg will miss many true cross-links. Nonetheless, we are adding a feature in the search application that will allow the user to limit the reactivity profile to any configuration he/she chooses.

<u>The following paragraph was added to the Results (line 127):</u>
“The modified +12 Da fragments in the MS/MS spectra allowed us to better characterize which amino acids are most likely to be cross-linked by the 24 Da reaction. To that end, we computationally modified in turn each residue along the cross-linked peptides, and determined which modification site was most compatible with the observed fragmentation pattern. The number of times each

amino acid was found to be the most compatible was then normalized by dividing it by the total number of occurrences of that amino acid. This analysis clearly marks lysine and arginine residues to be the most prevalent in the 24 Da reaction (Fig. S5). The high reactivity of formaldehyde with these two amino acids is fully consistent with previous studies performed on peptides and single amino acids (5, 7, 10). However, we note that a third of the identified cross-links involve at least one peptide that does not have a lysine residue. In these particular peptides aspartic acid and tyrosine residues are the most likely to be the linked residues. Interestingly, tyrosine was previously shown to be the third most reactive residue towards formaldehyde under certain conditions (5). We conclude that the majority of formaldehyde cross-links occur between lysine or arginine residues, but a significant fraction of cross-links also involve asparagine, histidine, aspartic acid, tyrosine, and glutamine residues."

The following figure was added (Fig. S5, line 784):

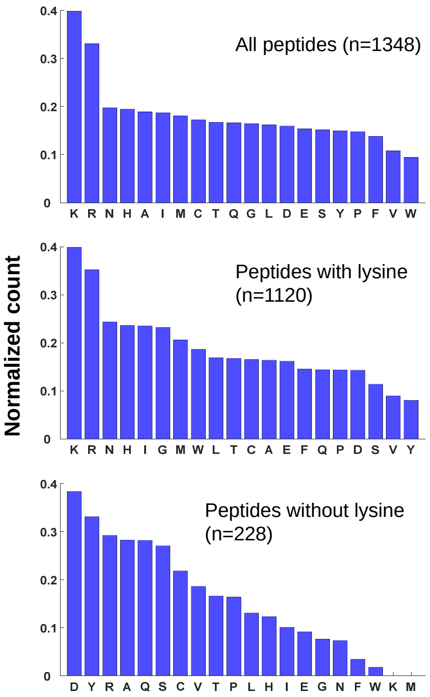

Figure S5. The propensity of different amino acids to participate in the 24 Da cross-link. For each peptide in each cross-link, we marked the most likely link site as the residue on which a 12 Da modification would be the most compatible with the MS/MS fragmentation. The normalized count is the total number of times a certain amino acid type was the most likely link site divided by the total number of occurrences of the same amino acid in all the peptides. The analysis on all the peptides (top plot) was also repeated on two subsets: peptides that have at least one lysine residue (middle plot), and peptides that have no lysine residues (bottom plot).

We added the following paragraph to the Methods (line 373):

“The identified cross-links from all the replicates involving 2% and 4% formaldehyde cross-linking were pooled together for this analysis. For each cross-link, we analyzed the two peptides independently of each other. For each peptide, we computationally modified (added 12 Da) in turn to each residue. We then determined which residue position was most compatible with the MS/MS fragmentation pattern (highest number of fragments that can be assigned by the modified peptide at 6 ppm tolerance). The number of times each amino acid was found to be the most compatible was then normalized by dividing it by the total number of occurrences of that amino acid in all the peptides (normalized count).”

3) The methods section does not mention the collision parameters for the HCD. Definitely add this, but also a study optimizing this should be added. In particular, is it possible at low energy to preferentially cleave the crosslink but leave the peptides largely intact for MS3 targeting. If the author have access to an ETD enabled instrument, I would also be interested in seeing whether the FA bridges are dissociated by electron transfer.

We thank the reviewer for spotting our remiss to report this important experimental parameters. We have done an optimization of the collision energy along with the original measurements. The optimal NCE was found to be 26. Very interestingly, the fragmentation behavior is very consistent with the suggestion of the reviewer!

The following text was added to the Results (line 108):

“We find additional evidence for this two-step fragmentation model when we follow the change in fragmentation as a function of the normalized collision energy (Fig. S4): Low collision energies are sufficient to break the cross-links, but are insufficient to break the stronger bonds for b- and y-fragments.“

The following figure was added (Fig. S4, line 771):

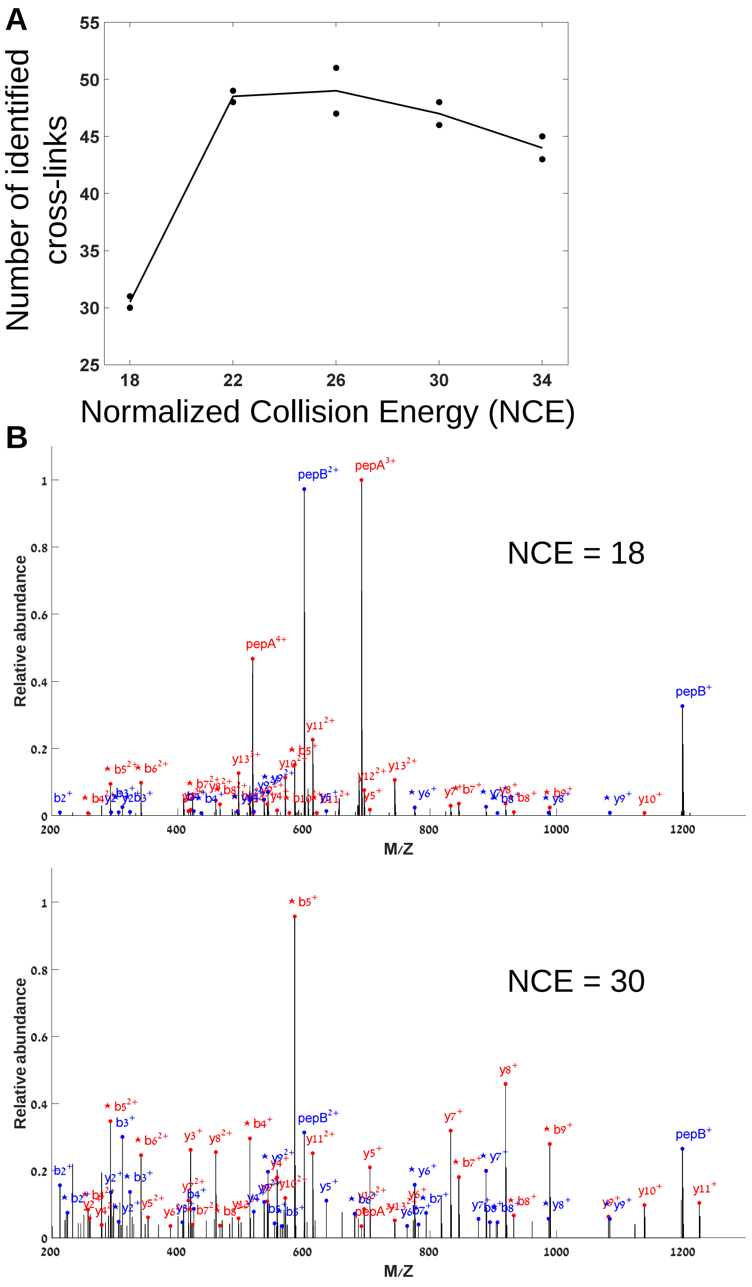

Figure S4. Effects of the Normalized Collision Energy (NCE). (A) The optimal NCE for identification of cross-links was determined to be 26. Shown are two experimental replicates (black spheres) measuring a series of HCD energies on a sample of 4% formaldehyde cross-linking of the three-protein mixture. A line through the average values is set to guide the eye. **(B)** Two MS/MS spectra of the same cross-linked ion consisting of CARNAPYSGYSGAFHCLK218 and DPVLKDLLFK320 from ovotransferrin, measured at two different NCE values. Note that the high peaks corresponding to the two intact peptides plus 12 Da (pepA and pepB) at NCE=18 are greatly reduced at higher NCE.

<u>The following text was added to the Methods (line 350):</u>

“HCD energy (NCE) 26 ;”

minor -

A) The authors mention that there is no direct evidence for observation of methylene bridged crosslinks. While I don’t disagree with their general conclusions, (ie model peptide based reactions are a poor way to study this), I don’t strictly speaking think this is true. For instance, ref 5, finds reaction between model peptide and glycine that are mediated by FA. Table I in that reference shows that a mass difference of +87 Da (eg Gly + 12Da) is the most common adduct. However, same table also finds +99 Da adducts which they attribute to multiple methylene bridges, but are also consistent with the mechanism here.

In the Introduction we changed the sentence (line 54): “direct evidence to support this mechanism is **lacking**” to “direct evidence to support this mechanism is **sparse**”.

We remark that Ref 5 is in fact very consistent with our general findings. Please see Table 3 in Ref 5, in particular the ‘Conversion’ column. Primary amines and arginine show the highest reactivity towards formaldehyde **by far**. Both of these amino acids only show the +99 adduct (corresponding to the 24 Da here).

B) Their search software annotates product ions that have a +12 Da modification as B- and Y- ions (upper case). I would avoid this, since this nomenclature is already well established for Glycopeptide fragmentation.

The nomenclature of “B” and “Y” was replaced by “*b” and “*y” throughout the text and figures.

C) For the mass modification scans - are these scanned as integer Dalton values? General practice is to model the average peptide distribution (“averagine”) and add a mass defect based on this.

Yes, the scans were only on integer Dalton values. We used a rather permissive MS1 tolerance of 6 ppm. We reasoned that with an average mass of 3000 Da for two cross-linked peptides our absolute search tolerance was 3000 * 6e-6 = 0.018 Da, which should be enough to detect a small adduct of a few N,C,O,H atoms.

D) Authors should probably formally separate out searching for mis-annotated mono-isotopic masses and the crosslink search in the search engine. Searching for +24, +25, +26 is fairly awkward and could be handled by a neutral loss search for 1 or 2-Da.

We found that searching for +24, +25, and +26 masses gives us the highest algorithmic efficiency. This is of course invisible to the user. The final report for

the user does not mention “+25 Da”, but rather “+24 Da ; (mono-isotopic assignment error of +1)”.
